# The multi-CDK inhibitor dinaciclib reverses bromo- and extra-terminal domain (BET) inhibitor resistance in acute myeloid leukemia via inhibition of Wnt/β-catenin signaling

**DOI:** 10.1186/s40164-024-00483-w

**Published:** 2024-03-04

**Authors:** Alexander R. Marr, Madeline Halpin, Dominique L. Corbin, Yerdanos Asemelash, Steven Sher, Britten K. Gordon, Ethan C. Whipp, Shaneice Mitchell, Bonnie K. Harrington, Shelley Orwick, Samon Benrashid, Virginia M. Goettl, Vedat Yildiz, Andrew D. Mitchell, Olivia Cahn, Alice S. Mims, Karilyn T. M. Larkin, Meixao Long, James Blachly, Jennifer A. Woyach, Rosa Lapalombella, Nicole R. Grieselhuber

**Affiliations:** 1 Division of Hematology, Department of Internal Medicine, The Ohio State University, Columbus, OH USA; 2 Department of Pathology, Stanford University, Stanford, CA USA; 3 College of Veterinary Medicine, Michigan State University, Lansing, MI USA; 4 Department of Biomedical Informatics, The Ohio State University, Columbus, OH USA; 5 Leukemia Research Program, The Ohio State University James Comprehensive Cancer Center, Columbus, OH USA

**Keywords:** AML, Dinaciclib, PLX51107, Wnt signaling, β-catenin, Resistance, Combination therapy

## Abstract

Acute myeloid leukemia (AML) is a highly aggressive hematologic cancer with poor survival across a broad range of molecular subtypes. Development of efficacious and well-tolerable therapies encompassing the range of mutations that can arise in AML remains an unmet need. The bromo- and extra-terminal domain (BET) family of proteins represents an attractive therapeutic target in AML due to their crucial roles in many cellular functions, regardless of any specific mutation. Many BET inhibitors (BETi) are currently in pre-clinical and early clinical development, but acquisition of resistance continues to remain an obstacle for the drug class. Novel methods to circumvent this development of resistance could be instrumental for the future use of BET inhibitors in AML, both as monotherapy and in combination. To date, many investigations into possible drug combinations of BETi with CDK inhibitors have focused on CDK9, which has a known physical and functional interaction with the BET protein BRD4. Therefore, we wished to investigate possible synergy and additive effects between inhibitors of these targets in AML. Here, we describe combination therapy with the multi-CDK inhibitor dinaciclib and the BETi PLX51107 in pre-clinical models of AML. Dinaciclib and PLX51107 demonstrate additive effects in AML cell lines, primary AML samples, and in vivo. Further, we demonstrate novel activity of dinaciclib through inhibition of the canonical/β-catenin dependent Wnt signaling pathway, a known resistance mechanism to BETi in AML. We show dinaciclib inhibits Wnt signaling at multiple levels, including downregulation of β-catenin, the Wnt co-receptor LRP6, as well as many Wnt pathway components and targets. Moreover, dinaciclib sensitivity remains unaffected in a setting of BET resistance, demonstrating similar inhibitory effects on Wnt signaling when compared to BET-sensitive cells. Ultimately, our results demonstrate rationale for combination CDKi and BETi in AML. In addition, our novel finding of Wnt signaling inhibition could have potential implications in other cancers where Wnt signaling is dysregulated and demonstrates one possible approach to circumvent development of BET resistance in AML.

**Supplementary Information:**

The online version contains supplementary material available at 10.1186/s40164-024-00483-w.

## Background

Acute myeloid leukemia (AML) is a highly heterogenous and aggressive hematologic neoplasm that has poor long-term survival with traditional cytotoxic chemotherapy [1]. In the last decade, significant progress in the understanding of AML disease etiology combined with development of novel sequencing technologies has led to the development of well-tolerated targeted therapies. Unfortunately, prognoses for patients with AML remains poor, with a 5-year overall survival rate of only 25–30% [2]. Development of effective treatments for AML patients remains challenging due to the broad molecular heterogeneity of the disease, associated co-morbidities in elderly patients, rapid disease progression at time of relapse, and evolution of resistance to current targeted therapeutics [3, 4].

Bromodomain and extra-terminal domain (BET) family proteins, including BRD2, BRD3, and BRD4, play crucial roles in many cellular functions important to leukemogenesis such as super-enhancer function, transcriptional elongation, and cell cycle progression [5]. These activities have been reported across multiple mutational subtypes of AML, including NPM1 mutated, IDH2 mutated, core binding factor, 11q23 rearranged, inv (3), monosomy 7, ASXL1 mutated, and FLT3 mutated [5–12]. Therefore, BET proteins have become an attractive target for novel therapeutics that may have activity in a wide range of AML molecular subtypes. Multiple pan-BET inhibitors (BETi) have progressed into clinical trials, although none have achieved approval due to poor tolerability, toxicity, and short response durations [13]. Recently, the effect of BETi on T-cell-mediated antitumor immunity have demonstrated suppression of PD-L1 expression and inhibition of AML cell line growth, providing further rationale for continued development of BETi in AML [14]. We have previously reported the development of PLX51107, a novel, structurally distinct BETi with improved pharmacologic properties compared to other compounds within the class [15].

Prolonged BETi exposure has resulted in development of resistance in multiple pre-clinical models of AML [16–25] and remissions in clinical trials of BETi are often short lived [26]. Transient clinical responses and eventual resistance to single agent targeted therapies in AML remains a major challenge in the field. Accordingly, there has been intense interest in defining tolerable combinations of targeted therapies to improve clinical responses in AML and circumvent development of resistance. Multiple mechanisms of BETi resistance in hematologic malignancies and solid tumors have been reported, including upregulation of diverse pro-survival signaling pathways, increased BRD4 expression, and stabilization of BRD4 protein [18–21, 23–25, 27–31]. Across a broad range of AML subtypes, BETi resistance is primarily mediated by upregulation of the canonical Wnt/β-catenin signaling pathway [16, 17], suggesting inhibition of Wnt signaling could represent a novel strategy to overcome therapy emergent BETi resistance.

Synergistic activity of BET and cyclin dependent kinase (CDK) inhibitors have been reported in a variety of malignancies [22, 32–38], including AML [32, 34]. To date, much of the investigation on mechanisms of synergy has focused on CDK9, which has a known physical and functional interaction with BRD4 [39, 40]. However, most CDK inhibitors have activity against multiple CDKs and off-target effects on other kinases [41], suggesting other pathways may also be relevant to the observed synergistic effect of BETi and CDKi.

Dinaciclib is a multi-CDK inhibitor with activity in the low nanomolar range against CDK1, CDK2, CDK5, CDK6, CDK7, CDK9 and CDK12 [42–44]. In addition to its well-characterized effects on CDKs, dinaciclib also has off target effects on GSK3β [45], a known component of the canonical Wnt/β-catenin signaling pathway. GSK3β inhibition has pleiotropic, context specific effects on Wnt signaling. In the Wnt-off state, cytosolic GSK3β phosphorylates β-catenin, facilitating its ubiquitination and subsequent proteasomal degradation, so GSK3β inhibition is predicted to activate Wnt signaling [46]. However, during active Wnt signaling, a membrane associated isoform of GSK3β additionally phosphorylates the Wnt co-receptor LRP6, promoting its engagement with the scaffolding protein AXIN2, activation of downstream signaling, and stabilization of catenin [47, 48]. In this context, GSK3β inhibition impedes rather than promotes Wnt signaling. Given the known constitutive activation of Wnt signaling in AML across multiple molecular subtypes [49, 50],we therefore decided to investigate the effect of dinaciclib on Wnt signaling both alone and in combination with PLX51107.

Herein, we report combination activity of dinaciclib and PLX51107 in pre-clinical models of AML. Further, we demonstrate novel inhibition of Wnt signaling by dinaciclib in both BETi naive and resistant cells, which retain both dinaciclib sensitivity and BETi/dinaciclib combination effects. Ultimately, this data provides a rationale for combination therapy with BET inhibitors and dinaciclib as a strategy to circumvent the development of Wnt-mediated resistance in AML.

## Methods

### Chemicals

PLX51107 was kindly provided by Plexxikon or purchased from MedChemExpress. Dinaciclib, cytarabine, etoposide, IWR-1, BML-284, and CHIR-99,021 (laduviglusib) were obtained from MedChemExpress. All chemical compounds were dissolved in Dimethyl Sulfoxide (DMSO) to prepare a 10 mM stock solution and were stored at -80 C for long-term storage as recommended.

### Cell lines

MV4-11 and MOLM-13 AML cell lines were purchased from Leibniz Institute DSMZ-German Collection of Microorganisms and Cell Cultures (Braunschweig, Germany). HS5-GFP stromal cells were a kind gift of Dr. William Dalton (H. Lee Moffitt Cancer Center). MOLM-13 cells expressing luciferase were generously provided by Dr. Ramiro Garzon [51]. TCF/LEF luciferase reporter NIH 3T3 cells were purchased from Enzo Life Sciences and cultured in Dulbeco’s Modified Eagle Medium (Life Technology, Carlsbad, CA) with Growth Medium Concentrate (Enzo Life Sciences), 100 U/mL penicillin, and 100 µg/mL streptomycin (Life technology CA), as recommended by the vendor. AML cell lines were cultured in RPMI 1640 medium (Life Technology) supplemented with 10% fetal bovine serum (VWR), 2 mM L-glutamine, 100 U/mL penicillin, and 100 µg/mL streptomycin (Life Technology) and grown in a 37 C, 5% CO2 incubator. Cell lines were routinely tested for mycoplasma contamination. Cell line identity was verified using short tandem repeat (STR) profiling.

### Primary AML and CD34 + cord blood samples

AML patient samples and CD34 + cord blood cells were obtained from The Ohio State University Comprehensive Cancer Center Leukemia Tissue Bank under an Institutional Review Board-approved protocol with informed consent according to the Declaration of Helsinki. Following rapid thawing of cryovials, cells were tested for viability using trypan blue exclusion before experimental use. In cell proliferation assays using primary patient samples, 1 × 10^5^ leukemia cells were plated in 96-well plates supported with either (1) 5 × 10^3^ HS5-GFP cells or (2) collagen type 1 (Life Technologies) coated wells and media supplemented with 10 ng/mL each of human GM-CSF, SCF, FLT3-L and IL-3 (PeproTech). For immunoblots and gene expression analysis, at least 1 × 10^6^ cells per condition were cultured in collagen coated 6-well plates in cytokine supplemented media.

### Cell proliferation assays

Cells were plated in 96 well plates at 1 × 10^5^ cells per well. Drugs were added at various concentrations and incubated for 72 h unless stated otherwise, at which point MTS reagent [3-(4,5-dimethylthiazol-2-yl)-5-(3-carboxymethoxyphenyl)-2-(4-sulfophenyl)-2 H-tetrazolium] (Promega) was added and incubated at 37 C according to the manufacturer’s instructions. Absorbance at 490 nm was measured with a DTX plate reader system. For stromal co-culture experiments, leukemia cells were transferred to a new plate prior to reading absorbance.

### Apoptosis assays and flow cytometry

Following treatment with drugs or vehicle, AML cell lines were stained with mouse anti-human annexin V-fluorescein isothiocyanate and propidium iodide (BD Pharmingen) according to the manufacturer’s instructions. For co-culture experiments, AML and HS-5 cells were stained with antibodies against CD33 (APC-Cyanine7, Clone P67.6, BioLegend) or CD34 (Brilliant Violet 421, Clone 581, BioLegend). Data was collected using a Gallios or CytoFLEX Flow Cytometer (Beckman Coulter) and analysis was performed using Kaluza software (Beckman Coulter).

### Colony forming assays

For colony forming assays, primary AML cells or CD34 + cord blood cells were plated at optimal density (generally 10,000–20,000 cells/mL dependent on patient sample) in MethoCult H4035 Optimum without EPO (Stem Cell Technologies) with DMSO as vehicle control, 1 µM PLX51107, 0.1 µM dinaciclib, or combination, followed by incubation in a humidified chamber in a 37 C, 5% CO2 incubator. Colonies were counted after 14 days by the automated STEMvision™ Hematopoietic Colony Counter (Stem Cell Technologies).

### Immunoblotting

AML cells (MV4-11 and MOLM-13) were drugged for 24 h, followed by protein extraction using RIPA buffer (Cell Signaling Technology) supplemented with PMSF. Cell pellets were incubated in RIPA buffer with constant agitation for 30 min, followed by sonication. Protein lysate was quantified using Pierce™ BCA Protein Assay Kit (ThermoFischer Scientific). 30 µg of protein was loaded into a Mini-PROTEAN TGX Stain-Free Precast Gel (Bio-Rad) and ran at 120 V for 1–2 h. Blots were developed using a ChemiDoc Chemiluminescence Imaging System (Bio-Rad) or by the Odyssey CLX Fluorescence Imaging System (Li-COR). Antibodies used in the presented studies purchased from Cell Signaling Technology included total β-catenin (#8480S), phosphorylated β-catenin (#9561S), non-phosphorylated β-catenin (cat#8814S), and β-actin (#3700S). Antibodies purchased from Abcam include LRP6 (ab134146) and phosphorlated-LRP6 (ab76417).

### Wnt LEADING LIGHT reporter assay

Recombinant Wnt3a, DKK, and the LEADING LIGHT Wnt Reporter Assay Kit were purchased from Enzo Life Sciences and used per the manufacturer’s instructions. Briefly, 2.5 × 10^5^ cells per well were seeded in 96-well plates with assay medium. Following an overnight incubation in a 37 C, 5% CO2 incubator, stock solutions were added to media to achieve final concentrations of 200 ng/mL recombinant Wnt3a or 200 ng/mL DKK. Wells were additionally treated with either 100 nM dinaciclib, 1 µM PLX51107, or combination dinaciclib and PLX51107. After a 24-hour incubation, luciferase substrate was added to each well, followed by measurement of chemiluminescent signal after a 10-minute incubation. Results were analyzed according to the manufacturer’s instructions.

### RNA extraction and quantitative RT-PCR

Cells were collected and prepared using QIAzol Lysis Reagent, followed by extraction and preparation of total RNA using the miRNeasy Kit (Qiagen). Following RNA concentration measurement (nanodrop), cDNA was transcribed. Real-time quantitative PCR was performed on an Applied Biosystems ViAA7 instrument utilizing TaqMan gene expression assays. 18 S was used as a normalization gene because unlike GAPDH, its expression is not dependent upon RNA polymerase II, which is inhibited via dinaciclib’s effects on CDK9 [52]. Results were quantified as a fold change using the comparative threshold method.

### Animal studies

All animal studies were approved by The Ohio State University Institutional Animal Care and Use Committee (IACUC). NSG mice were purchased from Charles River Laboratories and C57/BL6 mice were obtained from The Jackson Laboratory. Animals were 6–8 weeks old at the start of experiments. Mice were engrafted via tail-vein with 1 × 10^5^ MOLM-13-luciferase cells and randomized 3 days later to receive vehicle (oral gavage and intraperitoneal injection), PLX51107 by oral gavage, dinaciclib by intraperitoneal injection, or both drugs. PLX51107 was prepared as previously described [15]. Dinaciclib was dissolved in 20% cyclodextrin in sterile water immediately prior to use. Mice were euthanized upon meeting early removal criteria (> 20% weight loss, hind limb paralysis, lethargy, anorexia) and tissues collected and fixed in 4% formalin in PBS for further analysis.

### Histopathology

Formalin-fixed tissues from mice were prepared and stained as previously described [53]. Slides were read by a trained veterinary pathologist (BH) who was blinded to treatment groups.

### Software

Synergy analyses were generated utilizing COMbenefit software. Colony forming assays were counted using the automated STEMvision™ Hematopoietic Colony Counter and associated analysis software (Stem Cell Technologies). Gene expression heatmaps were generated using the Pheatmap R package. All plots were created using GraphPad Prism 9. Illustrations were created using Biorender.

### Statistics

Statistical analyses were performed by Vedat Yildiz and the OSU Center for Biostatistics with Statistical Analysis Software, version 9.4 (SAS Institute Inc., Cary, NC, USA). A linear mixed- effects model was used to compare differences between groups. A student’s *t*-test was performed for pairwise group comparisons. Survival curves were estimated using the Kaplan-Meier method and curve differences between groups were assessed using the log-rank test. Bonferroni correction was used to conserve the overall type I error at α = 0.05 due to multiple comparisons.

## Results

### PLX51107 and dinaciclib demonstrate both single agent and combination activity in pre-clinical models of AML

**Fig. 1:**
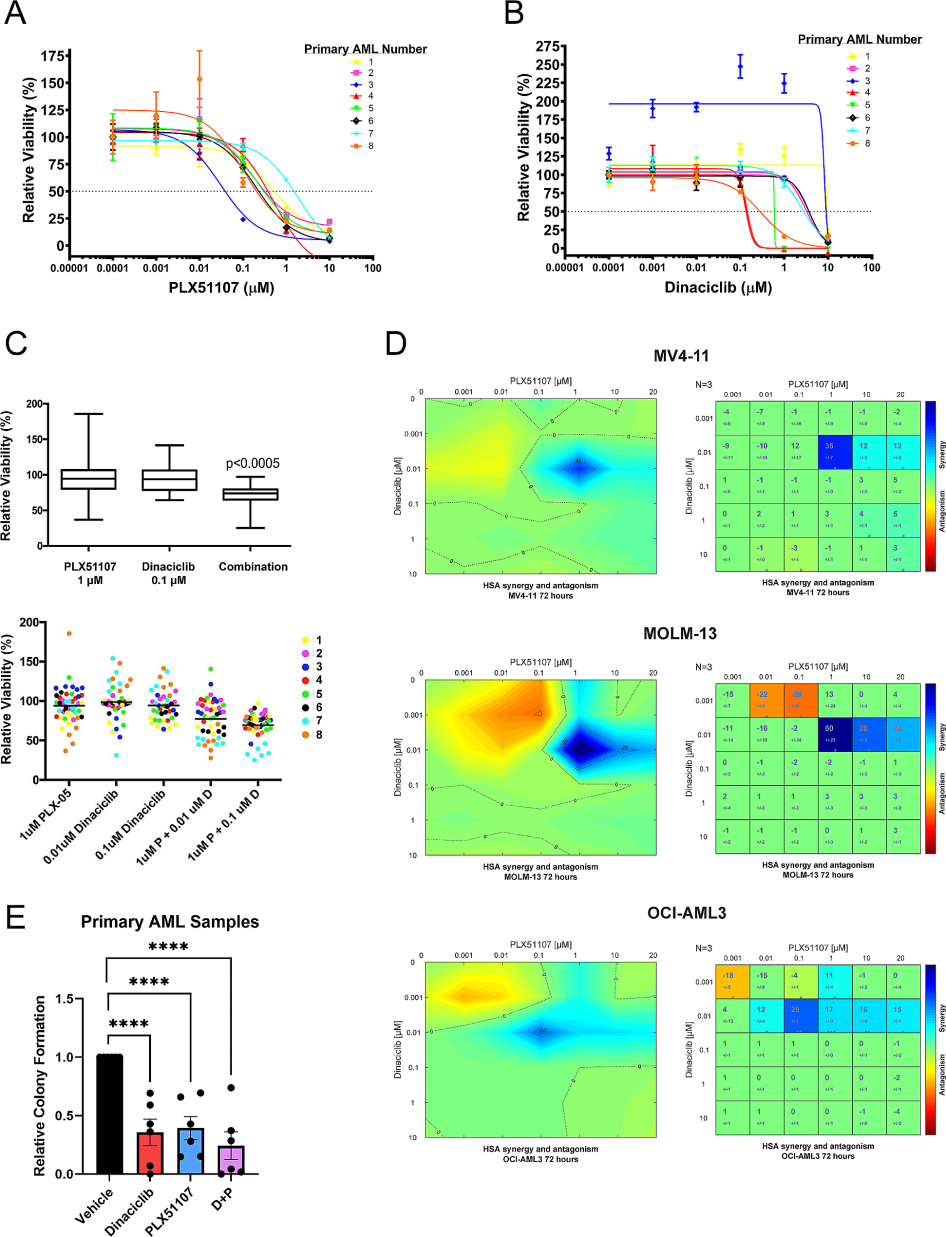
PLX51107 and dinaciclib demonstrate both single agent and combination activity in pre-clinical models of AML. (**A**) Cells from primary AML samples (*n* = 8) were co-cultured on HS-5 stroma in the presence of increasing concentrations of PLX51107. Relative viability was assessed using an MTS assay after 96 h. (**B**) Primary AML samples (*n* = 8) were treated with increasing concentrations of dinaciclib for two hours, followed by washout and plating with stroma. Viability was assessed using an MTS assay 94 h after co-culture. (**C**) The same primary AML samples (*n* = 8) were treated with 10 nM or 100 nM dinaciclib for 2 h, followed by washout and stromal co-culture with continuous exposure to 1 µM PLX51107 for 94 h (96 h total). Results are expressed as viability of combination treated cells relative to vehicle control (0.1 µM dinaciclib + PLX51107 vs. vehicle, *p* ≤ 0.0005). A mixed effects model, incorporating repeated measures for each patient sample was used for statistical analysis. (**D**) MV4-11, MOLM-13, and OCI-AML3 AML cell lines were treated with a range of doses of dinaciclib and PLX51107 for 72 h, and then, MTS reagent was added and absorption read. Highest single-agent (HSA) analysis was used to visualize regions of synergy. (*, **, *** = *p* ≤ 0.05, 0.01, and 0.001, respectively). (**E**) 10,000 cells/mL from primary AML samples (*n* = 6) were treated with vehicle control, dinaciclib (0.1 µM), PLX51107 (1 µM), or the combination and plated in triplicate in methylcellulose-based media. Colonies were counted after 14 days of incubation and expressed relative to vehicle treatment. (**** = *p* ≤ 0.0001)

Because many molecular subtypes of AML have been reported to be sensitive to BETi, we selected primary AML samples representing a wide range of mutations and cytogenetic abnormalities (Table 1) for in vitro investigations. Using a previously described HS-5 co-culture system [54], treatment of 8 primary AML samples with either continuous PLX51107 for 96 h (Fig. 1A) or a 2-hour exposure to dinaciclib followed by washout and stromal co-culture for 94 h (Fig. 1B) resulted in reduced viability of AML cells. Importantly, we found neither PLX51107 nor dinaciclib treatment affected stromal cell viability at our experimental doses and significant numbers of AML blasts were not left behind on the HS-5 layer after harvest (Supplemental Fig. 1A-B). We next investigated possible synergistic activity and additive effects of combination PLX51107 and dinaciclib by treating the same primary AML samples with a 2-hour exposure to dinaciclib that matches in vivo pharmacologic exposure previously reported in clinical trials [55, 56], followed by washout and stromal co-culture with continuous PLX51107 exposure for 94 h. Of note, continuous PLX51107 exposure in vitro more closely approximates the daily oral dosing used both in our previous pre-clinical work and in clinical trials [15, 57]. Here, we found a significant decrease in viability (Fig. 1C) by combination treatment with 0.1 µM dinaciclib and 1 µM PLX51107 when compared to vehicle control (*p* ≤ 0.0005, Supplemental Fig. 1C). Importantly, these concentrations of PLX51107 and dinaciclib have been previously achieved in vivo, both in pre-clinical studies and in clinical trials [15, 55–57].

**Table 1:**
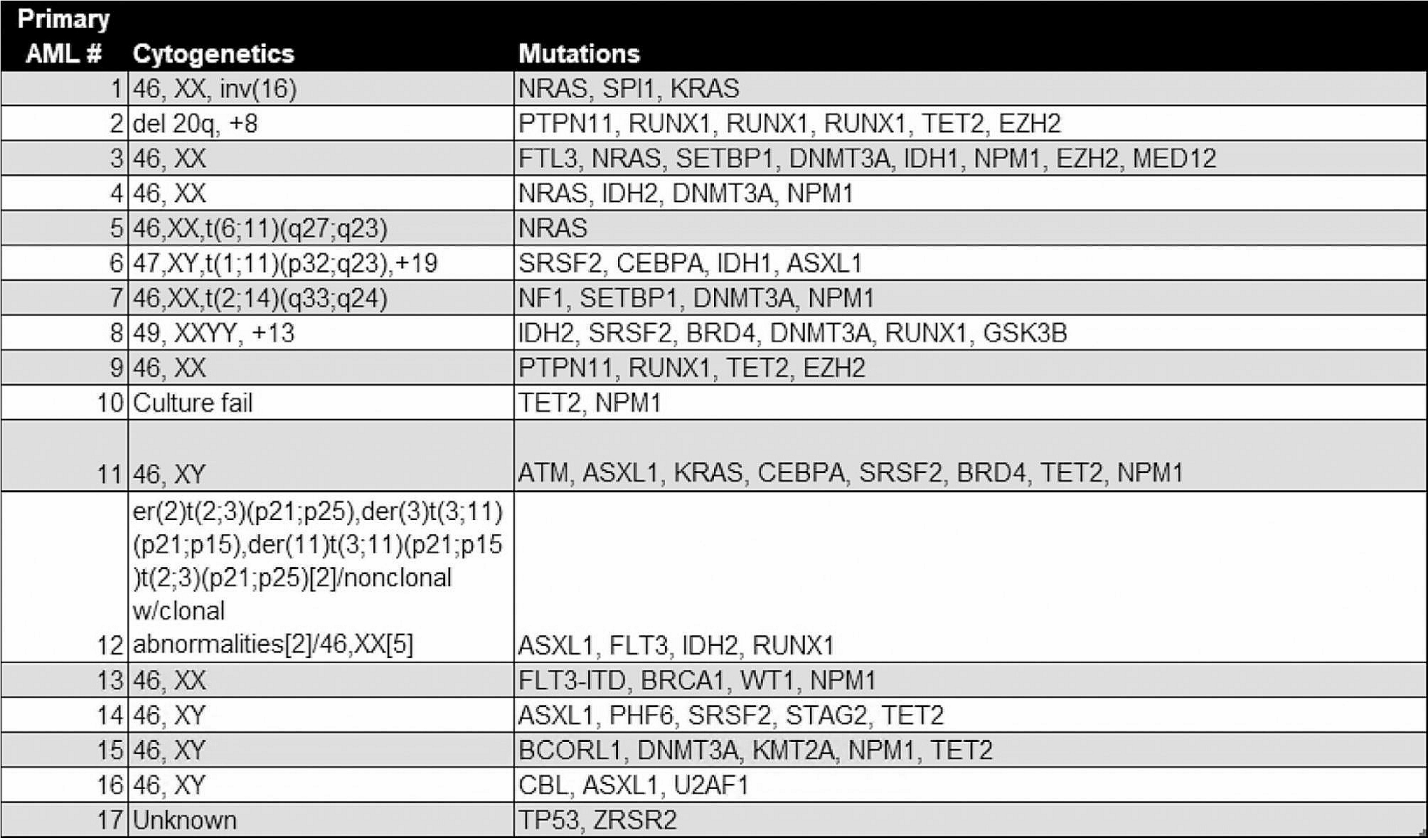
Primary AML samples used for in-vitro investigations

We further characterized possible synergistic activity of dinaciclib and PLX51107 by treating MV4-11, MOLM-13, and OCI-AML3 AML cell lines with several dose combinations. Utilization of COMbenefit synergy software allowed us to map levels of synergism across a range of doses to visualize patterns of synergy and observe dose combinations that exhibited the highest decrease in proliferation of AML cells (Fig. 1D). Synergistic activity was found across a range of PLX51107 and dinaciclib concentrations that are achievable in vivo [15, 55–57]. Additionally, we observed similar effects in primary AML cells cultured either with cytokine support or with HS-5 stromal co-culture, albeit at various concentrations due to the general heterogeneity of primary AML samples (Supplemental Fig. 1D and 1E). Moreover, we found combination PLX51107 and dinaciclib treatment produced a significant reduction in methylcellulose colony formation by multiple primary AML samples when compared to vehicle control (*p* ≤ 0.0001) and a more modest additive reduction when compared to either agent alone (Fig. 1E).

### Dinaciclib and combination treatment inhibits the canonical/β-catenin dependent Wnt signaling pathway at multiple levels in AML cells

**Fig. 2:**
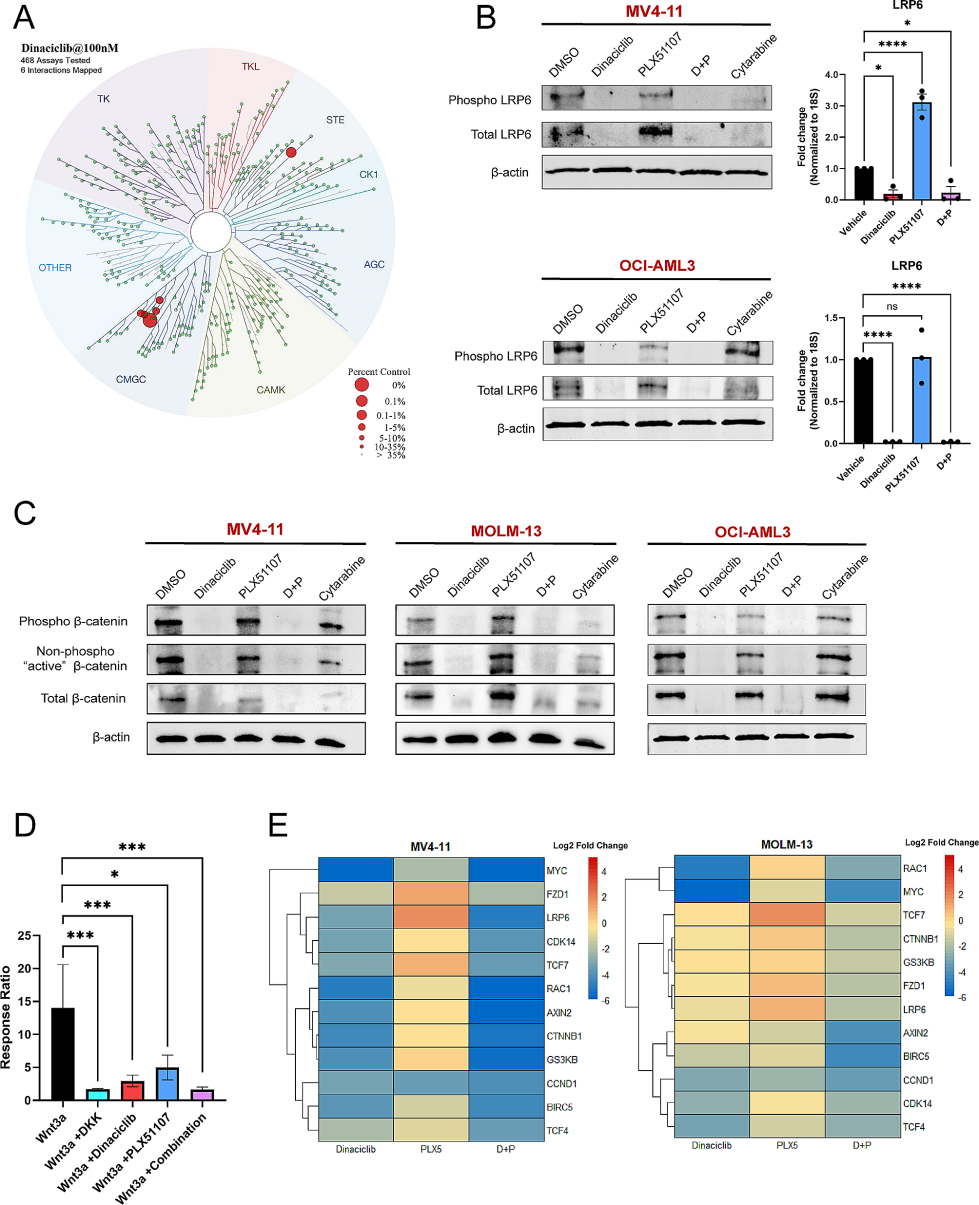
Dinaciclib and combination treatment inhibits canonical/β-catenin dependent Wnt signaling in AML cell lines. (**A**) Inhibitory activity of dinaciclib (100 nM) from the DiscoverX Kinome Scan was plotted in a representation of the human kinome. (**B**) Protein and mRNA expression of the Wnt co-receptor LRP6 following 24-hour treatment of MV4-11 and OCI-AML3 cells with DMSO as vehicle control, dinaciclib (0.1 µM), PLX51107 (1 µM), combination dinaciclib and PLX51107, or cytarabine (1 µM) (*n* = 3) (*, **** = *p* ≤ 0.05 and 0.0001 respectively). Dinaciclib washout and media replacement was performed at hour 4 in order to simulate in vivo exposure. (**C**) Representative immunoblots measuring protein expression of total, phosphorylated, and non-phosphorylated “active” β-catenin in MV4-11, MOLM-13, and OCI-AML3 AML cell lines. Cells were treated with DMSO as vehicle control, dinaciclib (0.1 µM), PLX51107 (1 µM), combination dinaciclib and PLX51107, or cytarabine (1 µM) for 24 h. (**D**) Functional Wnt activity assessed via the LEADING LIGHT Wnt reporter assay in which a luciferase reporter is under the control of a TCF/LEF binding site promoter. Results are from three independent biological replicates and shown as mean ± SEM. (*, *** = *p* ≤ 0.05 and 0.005 respectively). No significance in response was observed between induced Wnt activity in the presence of DKK versus dinaciclib, PLX51107, or combination treatment. (**E**) Summary of Wnt gene expression changes following 24-hour treatment with dinaciclib (0.1 µM), PLX51107 (1 µM), or combination dinaciclib and PLX51107. Changes are expressed as a log2 fold change relative to vehicle control

Since inhibition of GSK3β, a known dinaciclib target, may block Wnt signaling in the setting of constitutive activation [46], which is present in AML cells, we hypothesized that inhibition of Wnt signaling, a known BETi resistance mechanism [16, 17], may contribute to the observed combination activity. However, while dinaciclib has been reported to have off target effects on GSK3β [58], this activity was reported at higher concentrations than those at which synergy with PLX51107 was observed. Therefore, we decided to conduct kinome profiling of dinaciclib at 100 nM, a 10-fold lower concentration than previously reported to investigate other targets that may contribute to synergy with BETi (Fig. 2A). At this concentration, we observed little inhibition of GSK3β. As expected, dinaciclib potently inhibited its known CDK family targets at 100 nM. However, we also observed inhibition of CDK14, a member of the TAIRE family of cyclin dependent kinases, as well as TNIK, a member of the germinal center kinase family. Interestingly, CDK14 and TNIK play critical roles in activation and regulation of Wnt signaling. CDK14 promotes Wnt signaling via phosphorylation of the Wnt co-receptor LRP5/6 to activate the signaling cascade [59]. TNIK interacts directly with β-catenin and phosphorylates TCF4, leading to TCF/LEF-driven activation of Wnt target genes [60]. Therefore, we hypothesized dinaciclib treatment would inhibit canonical Wnt signaling.

To investigate the effects of dinaciclib on Wnt signaling, we first examined phosphorylated and total protein levels of LRP6. As predicted, dinaciclib treatment reduced phosphorylated and total LRP6 protein in MV4-11 and OCI-AML3 AML cell lines (Fig. 2B). Interestingly, PLX51107 treatment increased total LRP6 protein levels in MV4-11 cells, while combination treatment reduced LRP6 protein. We observed similar effects on mRNA expression of LRP6 as measured by RT-qPCR, with a significant decrease in dinaciclib and combination treated MV4-11 and OCI-AML3 cells (*p ≤* 0.05 and *p* *≤* 0.0001, respectively) and a significant increase in expression in PLX51107 treated MV4-11 cells (*p* ≤ 0.0001) when compared to vehicle control (Fig. 2B). After our initial findings of LRP6 inhibition by dinaciclib, we wished to further assess the effect of dinaciclib and possible synergistic activity of dinaciclib/PLX51107 on other components of the Wnt signaling pathway. We found dinaciclib and combination treatment of MV4-11, MOLM-13, and OCI-AML3 AML cell lines decreased total, phosphorylated, and non-phosphorylated “active” β-catenin protein levels as measured by immunoblots (Fig. 2C). As a control, we compared the observed inhibitory effects of dinaciclib treatment with a previously reported pharmacologic Wnt signaling antagonist, the tankyrase inhibitor IWR-1 [61–63]. IWR-1 modestly reduced mRNA expression of LRP6, TCF4, TCF7 and AXIN2 and had little effect on remaining genes examined (Supplemental Fig. 2A). Dinaciclib more potently reduced expression of Wnt pathway components and targets. Additionally, while IWR-1 treatment reduced protein levels of phosphorylated LRP6, total LRP6, total β-catenin and non-phosphorylated “active” β-catenin, the reduction was greater with dinaciclib treatment (Supplemental Fig. 2B). Therefore, we confirmed dinaciclib has activity on known targets of pharmacologic Wnt signaling inhibition.

Since it has previously been reported that BETi treatment of AML cells leads to transcriptional upregulation of Wnt signaling pathway components [16, 17], we next examined mRNA expression of CTNNB1, the gene encoding for β-catenin, as well as the Wnt receptor FZD1, CDK14, GSK3β, AXIN2, and the TCF/LEF transcription factors TCF4 and TCF7 in MV4-11, MOLM-13, and OCI-AML3 cell lines following treatment with dinaciclib, PLX51107, or the combination. We observed mRNA expression of Wnt signaling component genes was significantly decreased by dinaciclib or combination treatment when compared to vehicle control (Fig. 2E and Supplemental Fig. 3A, 4 A, and 5 A). Dinaciclib treatment also resulted in reduced expression of canonical Wnt pathway gene targets such as CCND1 [62], BIRC5 [63] and AXIN2 [64], and the non-canonical target RAC1 [65] (Supplemental Fig. 3A, 4 A, and 5 A). As a control, we also measured mRNA and protein expression of MYC, whose expression we and others have previously shown to decrease after CDK9 inhibition [66]. Consistent with previous findings, dinaciclib or combination treatment significantly reduced MYC mRNA and protein levels (Supplemental Fig. 3B and 4B) [15, 16].

In order to determine if Wnt signaling is functionally impaired by dinaciclib, we next utilized a well characterized Wnt reporter NIH 3T3 cell line in which luciferase cDNA is under the control of a LEF1/TCF binding site containing promoter. As expected, treatment of reporter cells with Wnt3a induced luciferase activity, indicating active Wnt signaling, while co-treatment with the well-characterized Wnt antagonist DKK [67] abrogated luciferase activity (*p* = 0.002) (Fig. 2D). Treatment of Wnt3a induced reporter cells with dinaciclib significantly reduced luciferase activity, similar to the effects of DKK, suggesting dinaciclib functionally inhibits TCF dependent transcription downstream of activated Wnt/β-catenin signaling (*p* = 0.005) (Fig. 2D). Reduction in luciferase activity was also observed after combination PLX51107 and dinaciclib treatment when compared to vehicle Wnt3a induced cells (*p* = 0.002) (Fig. 2D). No significant difference in luciferase expression was found between our treatment groups and DKK. Importantly, dinaciclib or PLX51107 treatment resulted in only a modest reduction in viability of the NIH 3T3 reporter cell line at 72 h of continuous drug exposure, suggesting the observed effects on Wnt signaling during the shorter 24-hour assay are not due to cytotoxic effects (Supplemental Fig. 6A). Additionally, dinaciclib reduced protein levels of the phosphorylated-serine 2 subunit of RNA polymerase II, a known effect of CDK inhibitors (Supplemental Fig. 6B), indicating that the drug is biologically active in these cells. Collectively, these results support that dinaciclib inhibits the Wnt/β-catenin signaling pathway at multiple levels in AML.

### Canonical Wnt signaling is inhibited by dinaciclib and combination treatment in primary AML samples

**Fig. 3:**
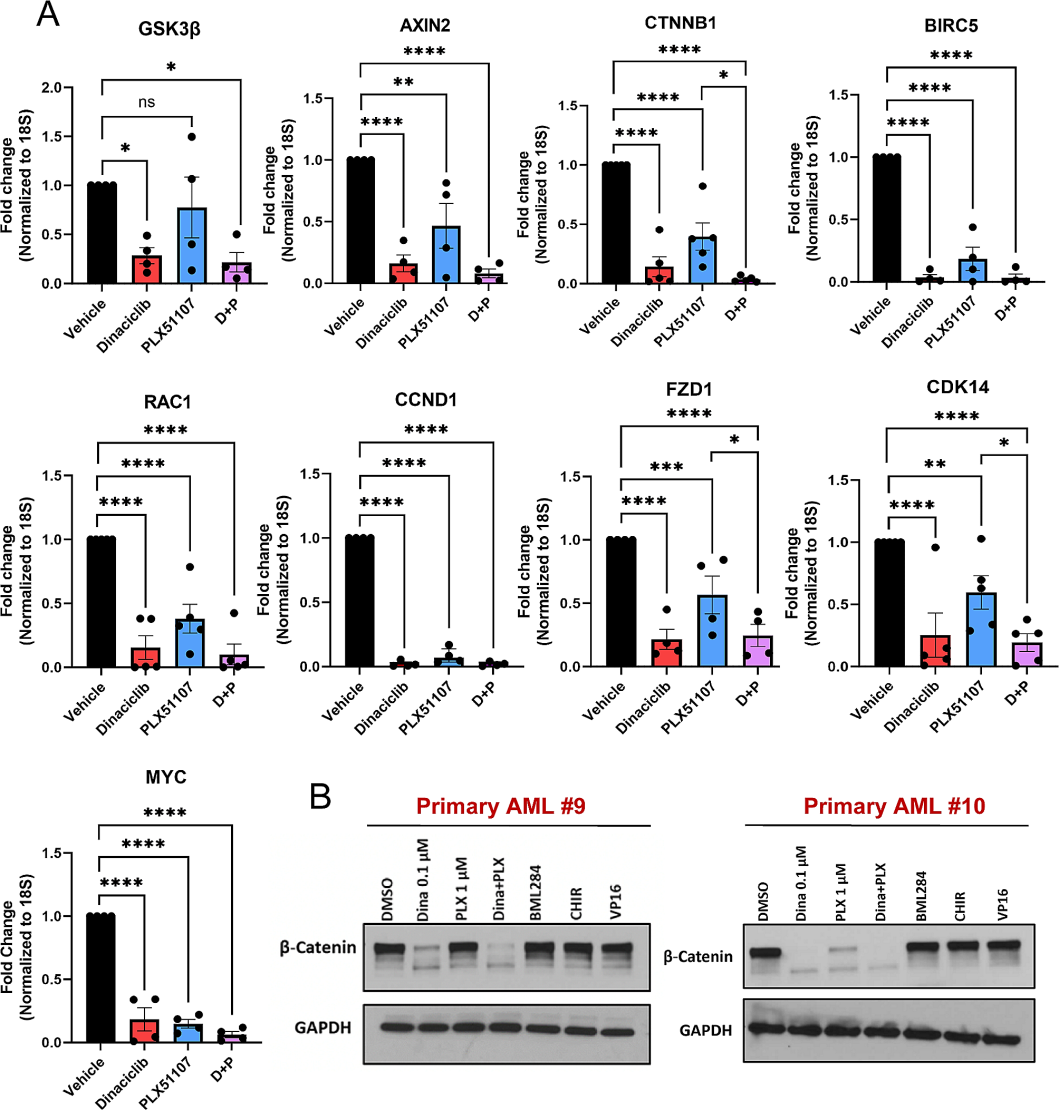
Canonical Wnt signaling is inhibited by dinaciclib and combination treatment in primary AML samples. (**A**) mRNA expression of Wnt components GSK3β, AXIN2, CTNNB1, FZD1, and CDK14, canonical targets BIRC5, CCND1 and MYC, and the non-canonical target RAC1 in primary AML cells (*n* = 4–5 per gene) as measured by RT-qPCR. Results shown as mean ± SEM. (*, **, ***, **** = *p* ≤ 0.05, 0.01, 0.005, and 0.0001 respectively). (**B**) Immunoblots measuring total β-catenin protein levels from two primary AML samples (#9 and #10) after treatment with DMSO as vehicle control, dinaciclib (0.1 µM), PLX51107 (1 µM), combination dinaciclib and PLX51107, BML284, CHIR, or VP-16 (1 µM) for 24 h. BML284 and CHIR were chosen as positive activators of Wnt signaling and VP-16 was used as a standard cytotoxic agent

As myeloid leukemia cell lines can have divergent biology from primary AML samples, we also examined mRNA expression changes of Wnt targets and components in primary AML samples following dinaciclib, PLX51107, or combination treatment, obtaining similar results to our cell lines (Fig. 3A). Interestingly, significance between single agent versus combination treatment was only achieved in PLX51107 treated cells, further suggesting the observed effects on Wnt signaling are strictly due to dinaciclib treatment. Furthermore, we found that dinaciclib or combination markedly reduced total β-catenin protein in primary AML cells (Fig. 3B). This was not seen in cells treated with pharmacologic activators of Wnt signaling (BML284, CHIR) [68] or with the cytotoxic agent etoposide (VP-16).

As an exploratory analysis, we examined mRNA expression of Wnt signaling components from the same primary samples utilized for our co-culture studies in Fig. 1A-B to explore possible associations between baseline expression and single agent response. As expected, we observed high expression of CTNNB1 in all examined AML samples (Supplemental Fig. 6C), indicative of active Wnt signaling as previously reported in AML [69–71]. We did note that the sample with the lowest CTNNB1 expression was also the least sensitive to dinaciclib (Supplemental Fig. 6D). Additionally, we did not observe significant differences in expression of GSK3β between our primary samples (Supplemental Fig. 6C), suggesting this target of dinaciclib is not directly responsible for observed differences in sensitivity between our primary samples. While the number of samples is insufficiently powered for statistical analysis, we did observe a trend towards higher LRP6 expression and lower dinaciclib IC_50_. Of the 3 samples with lowest dinaciclib IC_50_ (samples 2, 4 and 8), two of these (2 and 8) had high measured LRP6 expression. Conversely, the two samples with the highest dinaciclib IC_50_ (samples 6 and 7) had relatively low LRP6 expression. This trend was not present in all samples; for example, samples 3 and 4 were highly sensitive to dinaciclib but had low LRP6 expression, suggesting that LRP6 is not the sole determinant of dinaciclib sensitivity. We observed similar trends when comparing expression of TCF7 and dinaciclib sensitivity. We did not find any consistent relationship between baseline Wnt pathway gene expression and PLX51107 sensitivity. This is consistent with previously published reports showing Wnt signaling mediates BETi resistance via transcriptional rewiring that occurs with prolonged drug exposure [16, 17].

### BET resistant and less sensitive AML cells retain both dinaciclib sensitivity and dinaciclib / PLX51107 combination effects

**Fig. 4:**
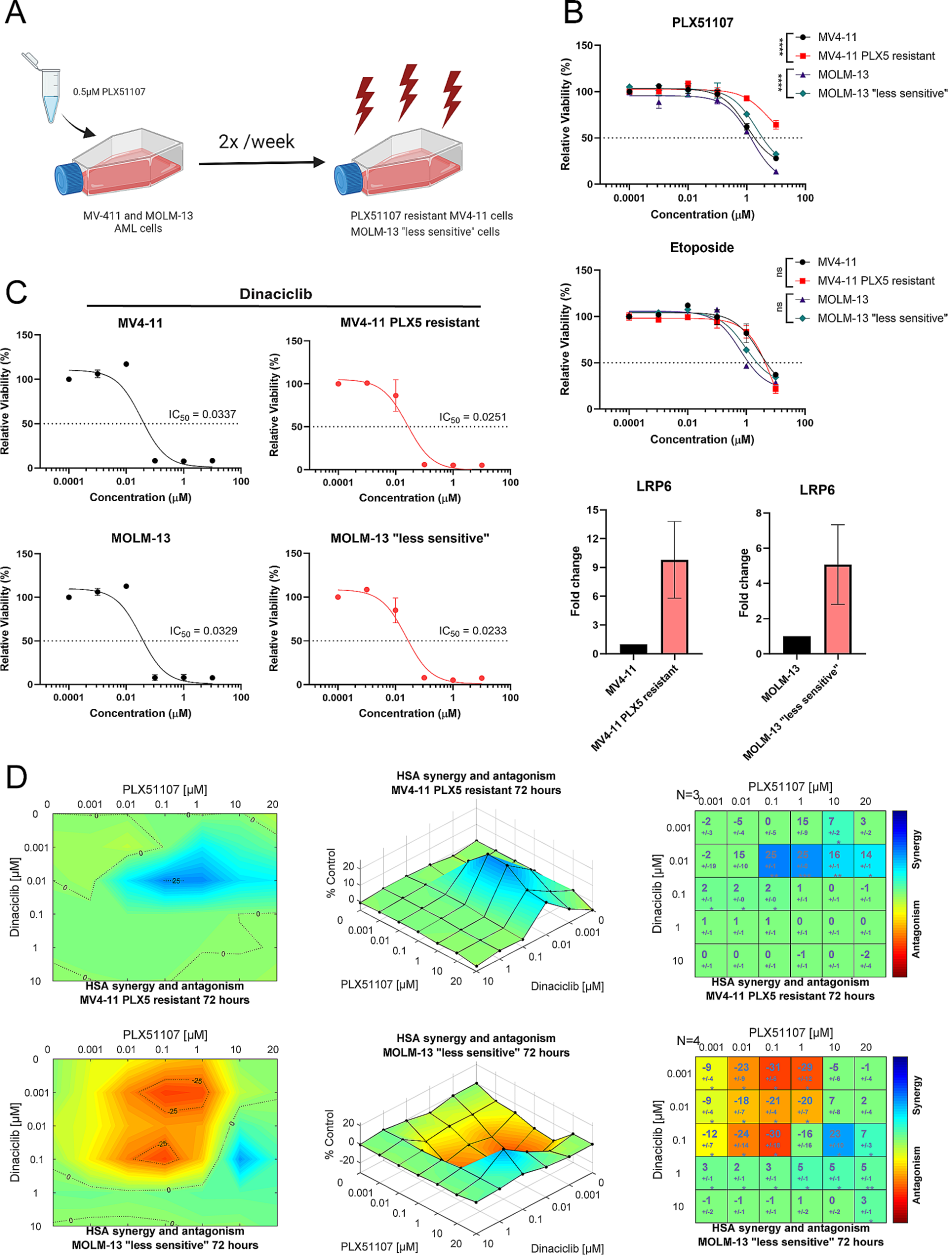
BETi resistant and less sensitive AML cells retain both dinaciclib sensitivity and dinaciclib / PLX51107 combination effects. (**A**) MOLM-13 and MV4-11 AML cell lines were serially drugged with 0.5 µM PLX51107 followed by drug washout and recovery for 6 months. (**B**) Dose-response curves of parental MV4-11 and MOLM-13 versus PLX51107 resistant MV4-11 and less sensitive MOLM-13 generated cell lines following 72-hour treatment with increasing doses of PLX51107 and etoposide. mRNA expression of Wnt co-receptor LRP6 in PLX51107 resistant MV4-11 and MOLM-13 cell lines compared to parental as measured by RT-qPCR. (**C**) Dose-response curves measured by MTS in MV4-11, PLX51107 resistant MV4-11, MOLM-13, and less sensitive MOLM-13 cell lines exposed to increasing doses of dinaciclib for 4 h, followed by washout and recovery for 72 h. Results are from three replicates. (**D**) PLX51107 resistant MV4-11 and less sensitive MOLM-13 cell lines were treated with a range of doses of dinaciclib and PLX51107 for 72 h, and then, MTS reagent was added and absorption read. Highest single-agent (HSA) analysis was used to visualize regions of synergy

Since we found dinaciclib could inhibit Wnt signaling, a known mechanism of BETi resistance in AML, we next hypothesized that dinaciclib could restore BETi sensitivity in resistant cells. We therefore generated PLX51107 resistant AML cell lines via serial drugging in order to assess dinaciclib’s effects in the setting of BETi resistance (Fig. 4). MV4-11 and MOLM-13 AML cell lines were serially passaged in media with PLX51107 followed by drug washout and recovery in drug-free media for over 6 months (Fig. 4A). We found MV4-11 serially drugged cells to be resistant to PLX51107 by MTS as intended (Fig. 4B) (IC_50_ = 1.04, 4.95, parental MV4-11, resistant MV4-11, respectively), while MOLM-13 cells solely became less sensitive to PLX51107 and did not develop full resistance (Fig. 4B) (IC_50_ = 1.44, 2.16 µM, parental MOLM-13, less sensitive MOLM-13, respectively). Importantly, our PLX51107 resistant MV4-11 and less sensitive MOLM-13 generated cells retained sensitivity to cytotoxic chemotherapy drugs such as etoposide (Fig. 4B), indicating that cells were not globally resistant to anti-leukemic compounds. In addition, to validate the involvement of Wnt signaling in our PLX51107 resistant and less sensitive generated cell lines, we performed gene expression analysis of Wnt signaling components, observing upregulation of the Wnt co-receptor LRP6 compared to parental controls (Fig. 4B). We did not observe significant changes in expression of other examined Wnt pathway genes (data not shown). This is consistent with other previously published models of BETi resistance in AML, in which only select pathway members’ expression is altered [16, 17]. When treated with dinaciclib, PLX51107 resistant MV4-11 and less sensitive MOLM-13 cells had a similar decrease in viability (Fig. 4C) when compared with their parental counterparts. Additionally, when our generated PLX51107 resistant and less sensitive cells were treated with combination PLX51107 and dinaciclib, synergy was still present via COMbenefit analyses, albeit at higher concentrations of PLX51107 than in parental cell lines (Fig. 4D). Importantly, these concentrations have been physiologically achieved in both in pre-clinical studies and in clinical trials [15, 55–57].

### Dinaciclib and combination treatment inhibit wnt signaling in BET resistant and less sensitive AML cells

**Fig. 5:**
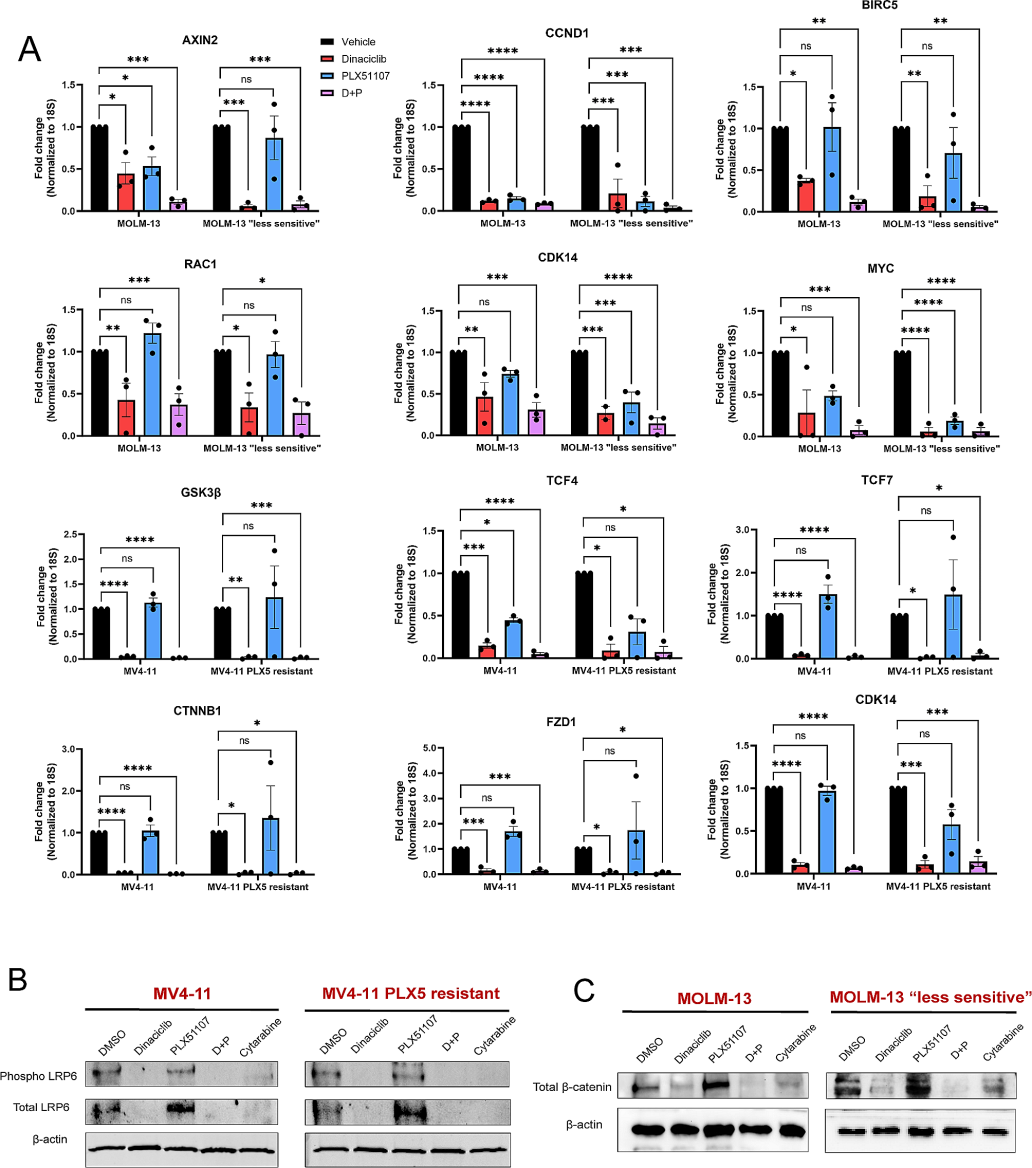
Dinaciclib and combination treatment continues to inhibit Wnt signaling in BETi resistant and less sensitive cells. (**A**) mRNA expression of Wnt components and targets in MOLM-13, MV4-11, PLX51107 resistant MOLM-13, and PLX51107 resistant MV4-11 AML cells following 24-hour treatment with DMSO as vehicle control, dinaciclib (0.1 µM), PLX51107 (1 µM), or combination. Results are shown as mean ± SEM. (*, **, ***, **** = *p* ≤ 0.05, 0.01, 0.005, and 0.0001 respectively). (**B**) Immunoblots measuring protein levels of phosphorylated and total LRP6 in MV4-11 and PLX51107 resistant MV4-11 cell lines following treatment with vehicle, dinaciclib (0.1 µM), PLX51107 (1 µM), combination, or cytarabine (1 µM) for 24 h. (**C**) Representative immunoblots of MOLM-13 and PLX51107 resistant MOLM-13 AML cells measuring protein levels of β-catenin following 24-hour treatment with DMSO as vehicle control, dinaciclib (0.1 µM), PLX51107 (1 µM), combination, or cytarabine (1 µM)

We then assessed dinaciclib’s inhibition of Wnt signaling in our BET resistant and less sensitive cell lines. Interestingly, components and targets of Wnt signaling continued to show significantly reduced mRNA expression after treatment with dinaciclib or the combination in our BETi resistant generated MV4-11 and less sensitive MOLM-13 cell lines (Fig. 5A). Moreover, protein levels of phosphorylated and total LRP6 (Fig. 5B) as well as total β-catenin (Fig. 5C) were still reduced following dinaciclib or combination treatment in our BETi resistant and less sensitive cell lines, similar to effects seen in the parental lines. Collectively, we show dinaciclib sensitivity and the additive effect of dinaciclib/BETi combination treatment is retained in both cells that are BET resistant as well as those who have not yet achieved full resistance, demonstrating one possible method to circumvent Wnt-mediated resistance in AML.

### Low dose dinaciclib + PLX51107 combination treatment prolongs overall survival and reduces disease burden in vivo while avoiding toxicity

To assess the effect of combination PLX51107 and dinaciclib treatment in an in vivo disseminated model of AML, we engrafted GFP/luciferase labeled MOLM-13 AML cells via tail vein injection into NOD/SCID IL2rγ−/− (NSG) mice, followed by initiation of PLX51107 20 mg/kg daily oral gavage, dinaciclib 30 mg/kg intraperitoneal injection 3 times weekly, combination, or vehicle treatment. PLX51107 and dinaciclib dosing and administration schedules were originally chosen based on previously published in vivo studies [15, 72]. Unexpectedly, the combination treated mice experienced early mortality (Supplemental Fig. 7A) beginning within days of drugging. There was no statistical difference in overall survival between vehicle and combination arm mice, with the survival of combination mice being only 16 days compared to 28 days for vehicle control mice. In contrast, animals receiving either drug as a single agent had significantly prolonged survival compared to vehicle controls, consistent with previous work [15, 72]. Histopathological analysis of combination treatment mice who met early removal criteria within 1 week of treatment initiation revealed microvesicular hepatic lipidosis and apoptosis of gastric and small intestine epithelium (Supplemental Fig. 7A). Importantly, there was no evidence of leukemic infiltration in any examined tissues, indicating that accelerated disease progression was not contributing to the early deaths. Of note, we had not previously observed these histopathologic changes in mice treated with PLX51107 alone in multiple hematologic malignancy models [15]. Because both BET proteins and CDKs are highly expressed in the liver, we hypothesized that the observed histopathologic changes reflected drug-induced hepatotoxicity. When the hepatocyte cell line HEP3B was treated with varying dose combinations of dinaciclib and PLX51107, there was a synergistic decrease in viability as measured by MTS assays (Supplemental Fig. 7B).

We hypothesized that a decreased dose and frequency of dinaciclib would be tolerated in combination with PLX51107 20 mg/kg. We therefore treated wild-type C57/Bl6J mice (*n* = 3 per dose) with 20 mg/kg daily PLX51107 combined with 4 different dinaciclib dose schedules: 15 mg/kg twice weekly, 15 mg/kg once weekly, 5 mg/kg twice weekly, and 5 mg/kg once weekly. At 2 weeks, mice were euthanized and subjected to histopathological analysis (Supplemental Fig. 7C). At the highest dinaciclib dose, one mouse died prior to study end but no animals treated on lower dose combinations died or met euthanasia criteria prior to 2 weeks. We found that mice treated with 15 mg/kg dinaciclib twice weekly in combination with PLX51107 had similar hepatic and gastrointestinal abnormalities as the NSG mice receiving 30 mg/kg dinaciclib three times weekly in our previous study (Supplemental Fig. 7C). In addition, marrow hypocellularity was present in mice treated with the two highest doses of dinaciclib. These changes occurred even in the absence of leukemia engraftment, indicating that the drug combination itself was responsible rather than an interaction of the combination with the leukemia cells in an in vivo system. Importantly, these gastrointestinal and hepatic abnormalities were not observed in mice treated with dinaciclib doses below 5 mg/kg. Moreover, PLX51107 in combination with 5 mg/kg dinaciclib weekly produced no histopathological abnormalities. Due to the observed marrow hypocellularity with combination treatment containing higher doses of dinaciclib, we examined colony formation of human CD34 + cord blood cells when treated with dinaciclib, PLX51107 or combination (Supplemental Fig. 7D). PLX51107 had no significant effect on CD34 + cell colony formation, while dinaciclib and combination treatment produced modest but statistically significant decreases when compared to vehicle. Of note, in the same colony forming assay, combination treatment resulted in a greater magnitude of reduction in primary AML colony formation (average fold change 0.35 compared to vehicle control) when compared to that reduction seen with CD34 + cells (average fold change 0.54 compared to vehicle control). In addition, while there was little variability between the responses of individual CD34 + cord blood samples to any treatment, some AML primary samples were exquisitely sensitive to combination treatment, with near complete abrogation of colony formation. This suggests that optimizing drug doses in combination therapy may achieve a “therapeutic window” in which AML cells are targeted while normal hematopoiesis is less affected. Based on these results, we selected dinaciclib 10 mg/kg weekly for further in vivo studies in combination with PLX51107. Of note, a weekly dinaciclib dosing schedule has been effectively utilized in clinical trials [56, 73].

**Fig. 6:**
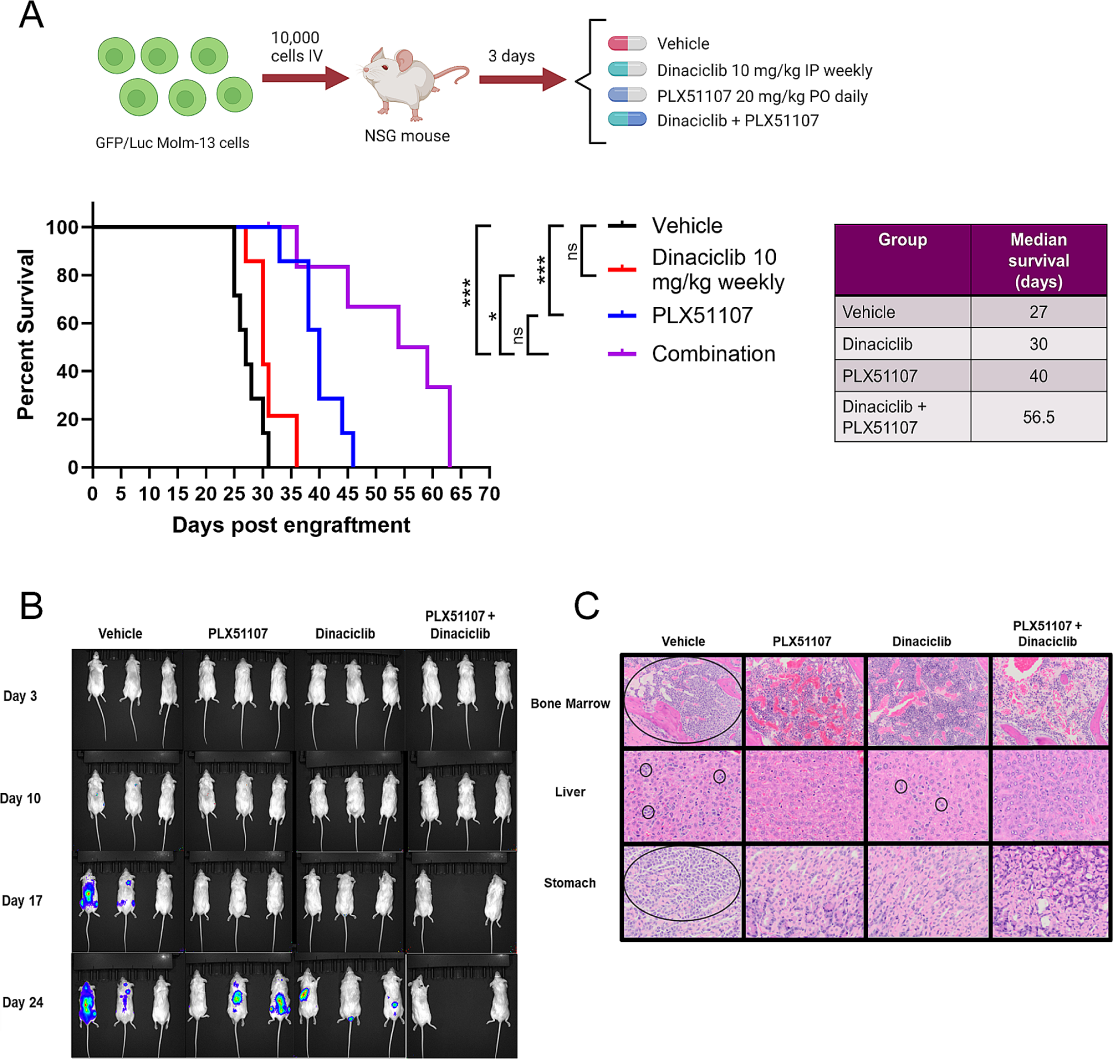
Dinaciclib and PLX51107 demonstrate additive effects and reduce disease burden in MOLM-13 xenograft mice. (**A**) 10,000 luciferase/GFP labeled MOLM-13 cells were xenografted by tail-vein injection in NSG mice, followed by a dosing schedule of PLX51107 20 mg/kg daily oral gavage, dinaciclib 10 mg/kg intraperitoneal injection once weekly, combination, or vehicle treatment (*, *** = *p* ≤ 0.05 and 0.005 respectively). (**B**) Weekly disease burden was assessed using a bioluminescent in-vivo imaging system (IVIS). Of note, one mouse in the combination arm died due to oral gavage error at week 2 and not due to leukemia burden or combination toxicity. (**C**) Histopathological analysis revealed the absence of previously observed microvesicular hepatic lipidosis and reduced infiltration of tissues in dinaciclib, PLX51107 and combination treatment arms when compared to vehicle treated mice

We next examined the efficacy of PLX51107 in combination with the new low-dose dinaciclib in vivo, again utilizing GFP/luciferase labeled MOLM-13 AML cells xenografted into NOD / SCID / IL2rg^null^ (NSG) immunodeficient mice (Fig. 6A). Consistent with our previous results [15], daily oral dosing with 20 mg/kg PLX51107 resulted in prolonged survival (median 42 days) compared to vehicle treated control animals (median 28 days, *p* = 0.0006). Low dose weekly dinaciclib alone did not significantly improve survival compared to vehicle treatment (median survival 30 days versus 27 days, *p* = 0.471), similar to results we have observed when investigating dinaciclib in combination with other targeted agents [54, 66]. However, mice receiving combination treatment experienced significantly increased survival compared to dinaciclib (*p* = 0.0072) or vehicle treated mice (*p* = 0.0021), with a median survival of 56.5 days. Of note, we did not observe any early lethality in combination low dose dinaciclib and PLX51107 treated mice. To investigate the effect of combination treatment on disease burden, a second cohort of mice were engrafted and treated as in Fig. 6A with planned euthanasia after 3 weeks of treatment. Bioluminescence imaging revealed decreased disease burden in combination treated animals when compared to vehicle control or either agent alone (Fig. 6B). Pathologic exam demonstrated no hepatic lipidosis or gastrointestinal epithelial apoptosis as well as decreased tissue infiltration by leukemic cells in the combination treatment animals, indicating both tolerability and efficacy of PLX51107 with a lower dose of dinaciclib (Fig. 6C). Ultimately, we demonstrated low dose dinaciclib and PLX51107 combination leads to increased survival and decreased tissue infiltration in vivo while avoiding toxicity.

## Discussion

We have demonstrated combination activity of the BET inhibitor PLX51107 with the multi-CDK inhibitor dinaciclib in AML cell lines, primary AML samples, and an in vivo model. Furthermore, combination treatment reduced primary AML cell colony forming activity, suggesting that this combination may target a leukemic stem cell population. Notably, both BRD4 and Wnt signaling have previously been shown to be important for maintenance of LSC populations [74].

Our study is the first to our knowledge to demonstrate inhibition of Wnt signaling by dinaciclib in AML. Wnt signaling was impacted at multiple levels following dinaciclib treatment, including reduced expression of the Wnt receptor FZD1, reduced expression of the Wnt co-receptor LRP6, reduced LRP6 phosphorylation, reduced expression of TCF family transcription factors, reduced “active” non-phosphorylated β-catenin, reduced CTNNB1 mRNA, and reduced total β-catenin protein. In addition, canonical (CCND1, BIRC5, AXIN2) and non-canonical (RAC1) Wnt target gene expression was decreased following dinaciclib treatment. Interestingly, dinaciclib treatment has also reduced β-catenin protein levels in pre-clinical ovarian cancer models [58], suggesting that inhibition of Wnt signaling by dinaciclib may be generalizable to other malignancies with activated Wnt signaling.

Importantly, we found BETi resistant MV4-11 and less sensitive MOLM-13 cells remain sensitive to dinaciclib and that dinaciclib continues to inhibit Wnt signaling in these models, a known BETi resistance mechanism in AML. In clinical trials, responses to BETi have been short lived [26]. This is not a challenge unique to BETi. With rare exceptions, single agent targeted therapies in AML do not produce durable complete remissions and development of resistance leading to relapse inevitably develops. Therefore, there has been great interest in developing novel combinations of targeted therapies to deepen responses, prolong durability of remissions, and overcome resistance, ultimately improving clinical outcomes. Our study provides justifiable rationale for combination treatment with a BETi and dinaciclib to overcome therapy emergent Wnt mediated resistance. In addition, Wnt signaling is known to promote resistance to other targeted therapies and chemotherapy both in AML and in other malignancies, so this strategy has implications beyond improving responses to BETi [75]. For example, chemoradiotherapy resistance in colorectal cancer cells has been shown to be mediated by Wnt/β-catenin signaling, suggesting dinaciclib could be of use for circumventing therapeutic resistance in other malignancies [76].

Surprisingly, there was unanticipated, lethal hepatotoxicity in mice treated with PLX51107 and dinaciclib using dose schedules which are tolerated as single agents in mice [15, 72]. While PLX51107 and dinaciclib share a common metabolism via CYP3A4, other investigations examining BET inhibitors for treatment of liver cancer suggest hepatotoxicity is not merely a result of drug-drug interaction but reflects normal role of their respective targets in hepatocyte biology where BET proteins have a known role in hepatocyte proliferation in response to injury [77–79]. We have previously shown that PLX51107 is more potent than first generation BET inhibitors such as JQ1, OTX015, and IBET-762 [15], so toxicity may also be related to drug potency, with more potent BETi perhaps being more likely to produce toxicity in drug combinations. In addition, CDK2, a dinaciclib target, regulates hepatocyte proliferation, both under steady-state physiologic conditions and in the setting of injury induced proliferation [80, 81]. It is also possible that the known dependence of hepatocytes and intestinal epithelium on Wnt signaling [77, 82]contributes to the observed toxicity. Of interest, we were able to determine a dose schedule of dinaciclib that could be given safely in combination with PLX51107 while still retaining an additive effect in combination with PLX51107. We further characterized synergy of our two compounds in relation to hepatocyte biology through COMbenefit synergy analysis on HEP3B cells, a common cell line derived from human hepatocellular carcinoma. Collectively, our results demonstrate that if combination BETi/CDKi are to be translated into early phase clinical studies, cautious dose escalation and close monitoring of liver function tests will be necessary. This is especially true in AML and other hematologic malignancies, where patients are often on concurrent medications (e.g. azole anti-fungals) with hepatotoxicity as a known potential adverse event. In addition, correlative pharmacokinetic studies could provide additional information on drug exposure, response, and toxicity.

Similarly, avoiding excess hematologic toxicity is paramount in AML patients, who are typically cytopenic as a result both of the malignancy itself and effects of treatment. We found that primary AML cells are more sensitive to PLX51107 / dinaciclib combination treatment in colony forming assays compared to healthy CD34 + cells (Supplemental Fig. 7D). This is consistent with work showing that BRD4 and β-catenin may be genetically abrogated in murine HSCs with only modest effects during steady state hematopoiesis and without inducing bone marrow failure [83, 84]. In addition, marrow hypocellularity did not develop in mice treated with low dose dinaciclib combination. These data provide evidence suggesting an acceptable therapeutic window for this combination.

The target or targets of dinaciclib mechanistically responsible for decreased Wnt signaling are unclear and deserving of further investigation. Dinaciclib is known to have off target effects on GSK3β and this initially prompted our investigation into its effect on Wnt signaling in AML. It is known that genetic deficiency of GSK3β in murine hematopoietic stem cells increases Wnt signaling and produces myelodysplasia, ultimately promoting leukemogenesis in combination with other cooperating events [85]. However, GSK3β has pleotropic context specific effects in the Wnt/β-catenin pathway and other signaling pathways. During active Wnt signaling, a membrane associated isoform of GSK3β phosphorylates the Wnt co-receptor LRP6, promoting formation of the Wnt signaling complex [47, 48]. Therefore, the effects of GSK3β inhibition may be quite different in fully transformed leukemic cells with constitutive Wnt signaling than in normal or pre-leukemic cells with more intermittent activation of this pathway. However, GSK3β inhibition occurs at higher concentrations than those at which synergy with PLX51107 was observed. At these lower concentrations, we did not find significant inhibition of GSK3β (Fig. 2A), suggesting that other dinaciclib targets are responsible for Wnt signaling inhibition.

Our group has previously examined the role of CDK9 in FLT3 mutated AML by performing RNA-sequencing analysis on MOLM-13 cells transduced with short hairpin RNA (shRNA) targeting CDK9 or scrambled as a control [66]. Upon GSEA analysis of shCDK9 vs. scrambled controls, we found modest downregulation of hallmark canonical Wnt signaling that was not statistically significant (Supplemental Fig. 8). This indicates that CDK9 inhibition alone is insufficient for significant reduction of hallmark canonical Wnt signaling. We found that dinaciclib inhibits TAIRE family kinases, including CDK14, and demonstrated that phosphorylation of LRP6, a known CDK14 target, is decreased by dinaciclib treatment, suggesting one possible mechanism. CDK14’s role in hematologic malignancies is relatively unexplored, especially compared to typical CDKs. It is also possible that other kinases inhibited by dinaciclib such as TNIK may have also be responsible for the observed effect on Wnt signaling. It has previously been shown that crosstalk between mitochondria and Wnt signaling provides an additional mechanism in a feedback loop where Wnt activation increases mitochondrial function that in turn drives more Wnt signaling [86]. Our group has shown that CDK9 inhibition reduces oxidative phosphorylation and fatty acid metabolism in AML [66]. It is possible that inhibition of CDK9 by dinaciclib may have indirect effects on Wnt signaling via altered mitochondrial function. Elucidation of the mechanism by which Wnt signaling is specifically inhibited by dinaciclib remains to be explored, both in situations with constitutively active Wnt signaling as well as more intermittent activation. Utilization of gene-editing techniques would provide further information about the precise role of these pathways in development of resistance and is a possible future direction of this work. Additionally, while our data in AML cell lines and primary AML cells cultured without stroma supports a cell intrinsic inhibition of Wnt signaling by dinaciclib, we did not examine the effect of dinaciclib or combination treatment on stromal expression of adhesion molecules or Wnt ligands. It is therefore possible in co-culture experiments and in vivo that Wnt signaling is also affected by treatment effects on the microenvironment. Of note, previous studies suggest that bone marrow stroma in AML may both promote Wnt signaling in leukemic stem cells [87] as well as inhibit cytotoxic T-cell mediated anti-leukemic responses in a Wnt dependent manner [88].

One limitation of our work is that the number of primary AML samples studied was insufficient for statistical power to determine associations between mutation or cytogenetic classification, Wnt signaling activation, and sensitivity to dinaciclib or combination treatment. Wnt signaling is a critical pro-survival pathway in multiple AML subtypes [49, 50], so it may be challenging to correlate mutations with sensitivity. Given that at present only a minority of AML patients have mutations that can be specifically targeted, there is great clinical utility in developing therapies that do not depend upon presence of a specific mutation. We did observe a trend towards higher expression of some Wnt signaling components, including LRP6 and TCF7, being associated with dinaciclib sensitivity in our patient samples. If this combination is investigated clinically, determining the relationship between Wnt pathway expression, activation, and clinical response will be an important correlative study. Of note, there are other drugs in clinical development for AML which use target protein expression rather than mutations as a predictive biomarker. For example, retinoic acid receptor alpha (RARA) positivity, which may be present in many mutational subtypes, is correlated with sensitivity to tamibarotene [89, 90]; there are on-going clinical trials of this agent in RARA expressing AML and MDS (NCT04905407 and NCT04797780).

A phase 1 clinical trial of dinaciclib in combination with venetoclax was recently completed in relapsed/refractory AML (NCT03484520), further highlighting dinaciclib’s ongoing development in hematologic malignancies and need for further characterization of its mechanistic properties. There are multiple CDK inhibitors in pre-clinical and early clinical development, each with their own spectrum of activities and off-target effects. We did not investigate the effect of other CDK inhibitors on Wnt signaling, many of which are in pre-clinical and early clinical development, each with their own spectrum of activities and off-target effects [91]. It is possible these other CDK inhibitors may have greater or lesser effects on Wnt signaling and mechanisms of Wnt inhibition by individual inhibitors remain to be defined. This is deserving of future investigation, especially given that despite many years of investigation, no drug specifically targeting Wnt signaling has yet been approved for any disease and repurposing drugs may represent a viable strategy to target an “undruggable” pathway.

## Conclusions

Collectively, our results provide pre-clinical support for further investigation of dinaciclib/BETi combination treatment in AML. We have described activity of combination PLX51107 and dinaciclib in primary AML cells and several cell line models. Our finding of unanticipated hepatotoxicity in vivo provides important information for the safe design of early phase clinical trials combining BETi and CDKi, both in AML and in other malignancies. Importantly, the combination was tolerable in vivo with dose reduction of dinaciclib while retaining synergistic activity, demonstrating feasibility. In addition, our novel finding of inhibition of Wnt signaling by dinaciclib may have implications for the treatment of other malignancies that are dependent upon Wnt signaling for survival. Finally, we have demonstrated that BETi resistant AML cells remain sensitive to dinaciclib, providing additional rationale for combinational therapy of compounds in these classes.

### Electronic supplementary material

Below is the link to the electronic supplementary material.

Supplementary Material 1

## Data Availability

No datasets were generated or analysed during the current study.

## Abbreviations

AML: Acute myeloid leukemia
ASXL1: Additional sex combs like-1
AXIN2: Axis inhibition protein 2
BCL2: B-cell lymphoma 2
BET: Bromo- and extra-terminal domain
BETi: Bromo- and extra-terminal domain inhibitor
BIRC5: Baculoviral inhibitor of apoptosis repeat containing 5
BRD2: Bromodomain containing 2
BRD3: Bromodomain containing 3
BRD4: Bromodomain containing 4
β-catenin: Beta-catenin
CCND1: Cyclin D1
CDK: Cyclin-dependent kinase
CDKi: Cyclin-dependent kinase inhibitor
CTNNB1: Catenin beta 1
DKK: Dickkopf-1
DMSO: Dimethylsulfoxide
FLT3: FMS-like tyrosine kinase 3
FZD1: Frizzled-1
GSK3β: Glycogen synthase kinase-3 beta
IDH2: Isocitrate dehydrogenase 2
IVIS: In-vivo imaging system
LRP6: Low density lipoprotein receptor-related protein 6
NPM1: Nucleophosmin 1
NSG: NOD/SCID gamma
RAC1: Rac family small GTPase 1
TCF/LEF: T-cell factor/lymphoid enhancer factor
TCF4: Transcription factor 4
TCF7: Transcription factor 7
