# Supplementary material for: The multi-CDK inhibitor dinaciclib reverses bromo- and extra-terminal domain (BET) inhibitor resistance in acute myeloid leukemia via inhibition of Wnt/β-catenin signaling
Source: Exp Hematol Oncol. 2024 Mar 4;13:27. doi: 10.1186/s40164-024-00483-w (PMC10913666; doi:10.1186/s40164-024-00483-w)
Supplement: Supplementary file 1 — Supplementary Material 1 [file 40164_2024_483_MOESM1_ESM.pptx]

## Slide 1
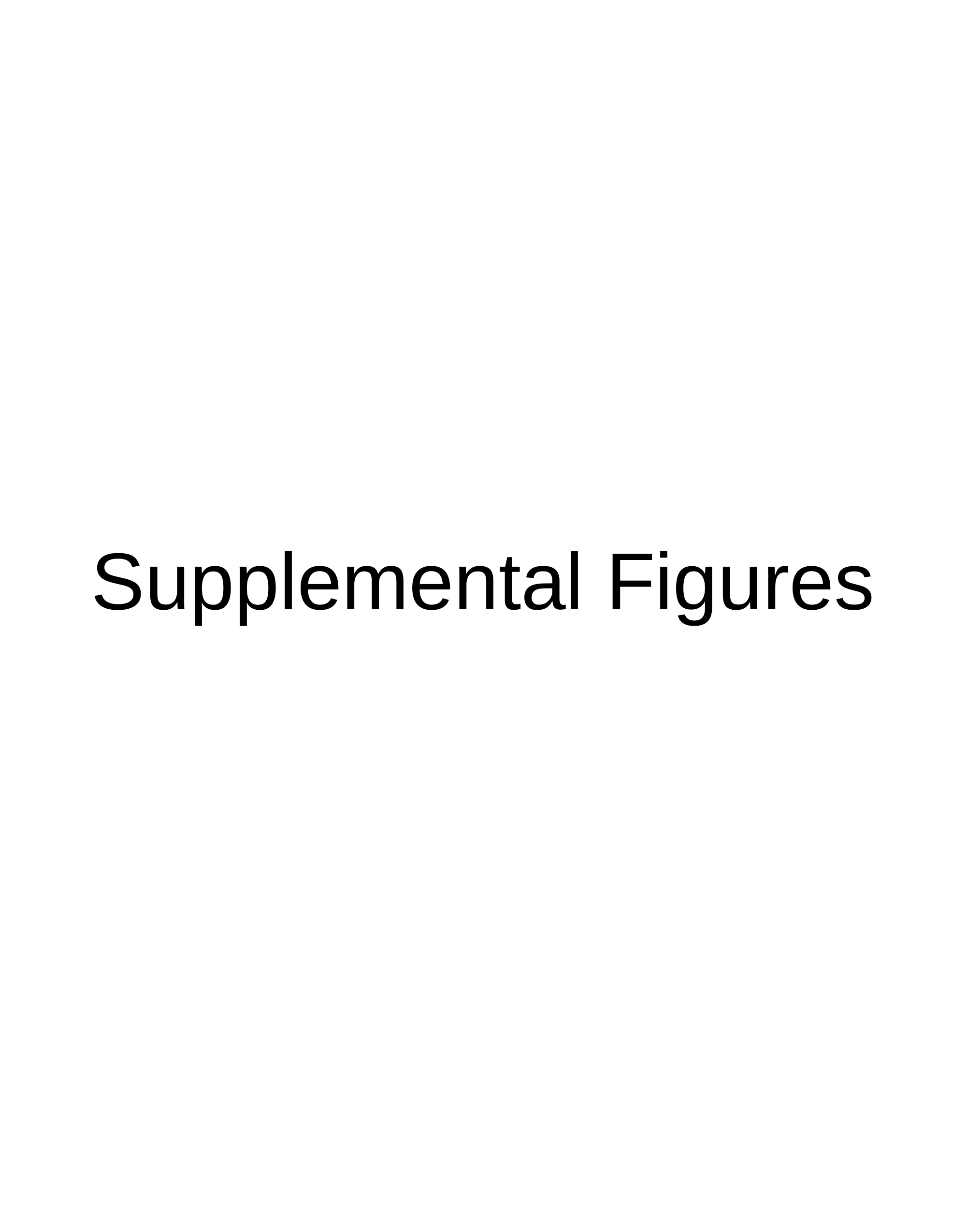

# Supplemental Figures

## Slide 2
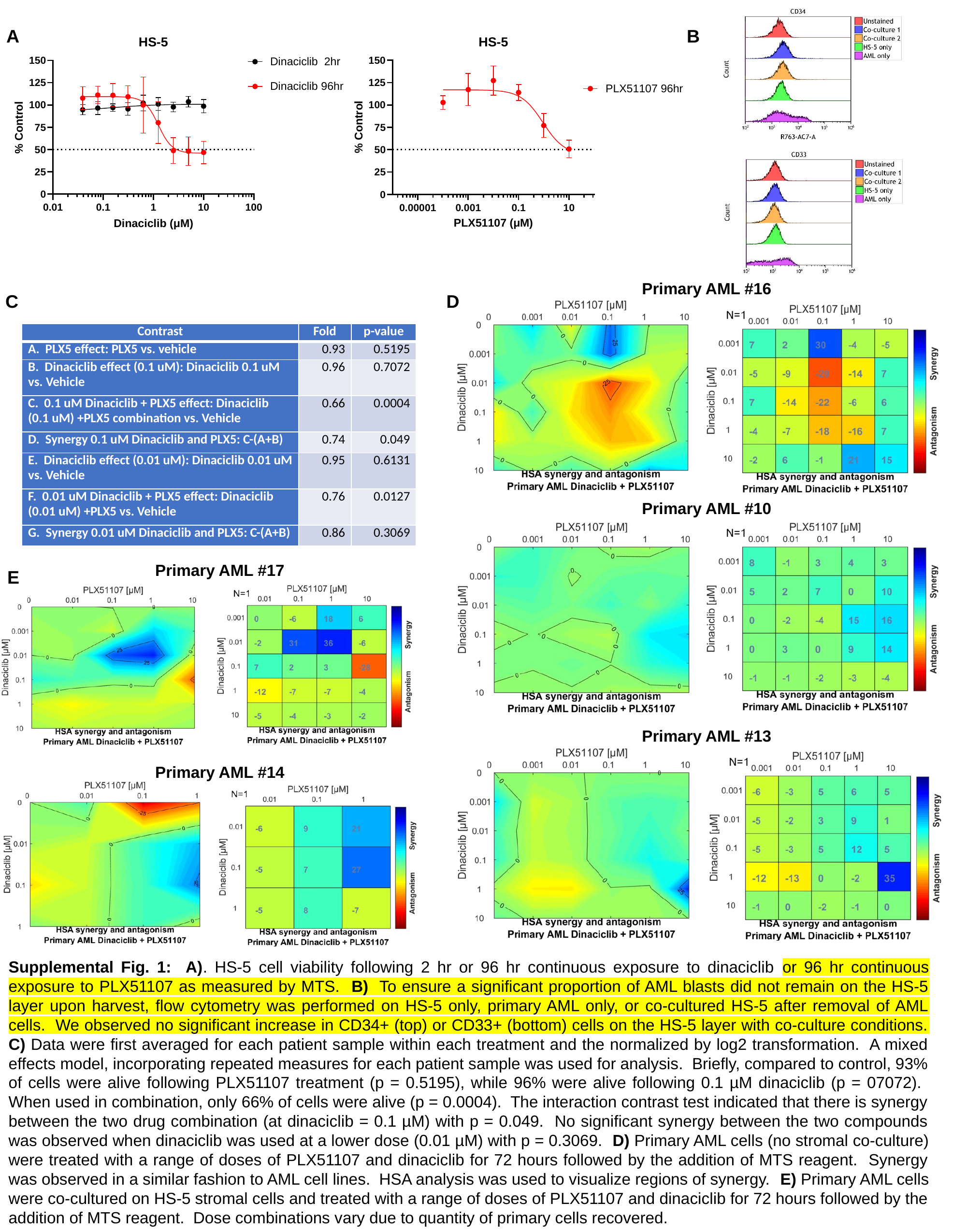

A
B
Primary AML #16
D
C
| Contrast | Fold | p-value |
| --- | --- | --- |
| A. PLX5 effect: PLX5 vs. vehicle | 0.93 | 0.5195 |
| B. Dinaciclib effect (0.1 uM): Dinaciclib 0.1 uM vs. Vehicle | 0.96 | 0.7072 |
| C. 0.1 uM Dinaciclib + PLX5 effect: Dinaciclib (0.1 uM) +PLX5 combination vs. Vehicle | 0.66 | 0.0004 |
| D. Synergy 0.1 uM Dinaciclib and PLX5: C-(A+B) | 0.74 | 0.049 |
| E. Dinaciclib effect (0.01 uM): Dinaciclib 0.01 uM vs. Vehicle | 0.95 | 0.6131 |
| F. 0.01 uM Dinaciclib + PLX5 effect: Dinaciclib (0.01 uM) +PLX5 vs. Vehicle | 0.76 | 0.0127 |
| G. Synergy 0.01 uM Dinaciclib and PLX5: C-(A+B) | 0.86 | 0.3069 |
Primary AML #10
Primary AML #17
E
Primary AML #13
Primary AML #14
Supplemental Fig. 1: A). HS-5 cell viability following 2 hr or 96 hr continuous exposure to dinaciclib or 96 hr continuous exposure to PLX51107 as measured by MTS. B) To ensure a significant proportion of AML blasts did not remain on the HS-5 layer upon harvest, flow cytometry was performed on HS-5 only, primary AML only, or co-cultured HS-5 after removal of AML cells. We observed no significant increase in CD34+ (top) or CD33+ (bottom) cells on the HS-5 layer with co-culture conditions. C) Data were first averaged for each patient sample within each treatment and the normalized by log2 transformation. A mixed effects model, incorporating repeated measures for each patient sample was used for analysis. Briefly, compared to control, 93% of cells were alive following PLX51107 treatment (p = 0.5195), while 96% were alive following 0.1 µM dinaciclib (p = 07072). When used in combination, only 66% of cells were alive (p = 0.0004). The interaction contrast test indicated that there is synergy between the two drug combination (at dinaciclib = 0.1 µM) with p = 0.049. No significant synergy between the two compounds was observed when dinaciclib was used at a lower dose (0.01 µM) with p = 0.3069. D) Primary AML cells (no stromal co-culture) were treated with a range of doses of PLX51107 and dinaciclib for 72 hours followed by the addition of MTS reagent. Synergy was observed in a similar fashion to AML cell lines. HSA analysis was used to visualize regions of synergy. E) Primary AML cells were co-cultured on HS-5 stromal cells and treated with a range of doses of PLX51107 and dinaciclib for 72 hours followed by the addition of MTS reagent. Dose combinations vary due to quantity of primary cells recovered.

## Slide 3
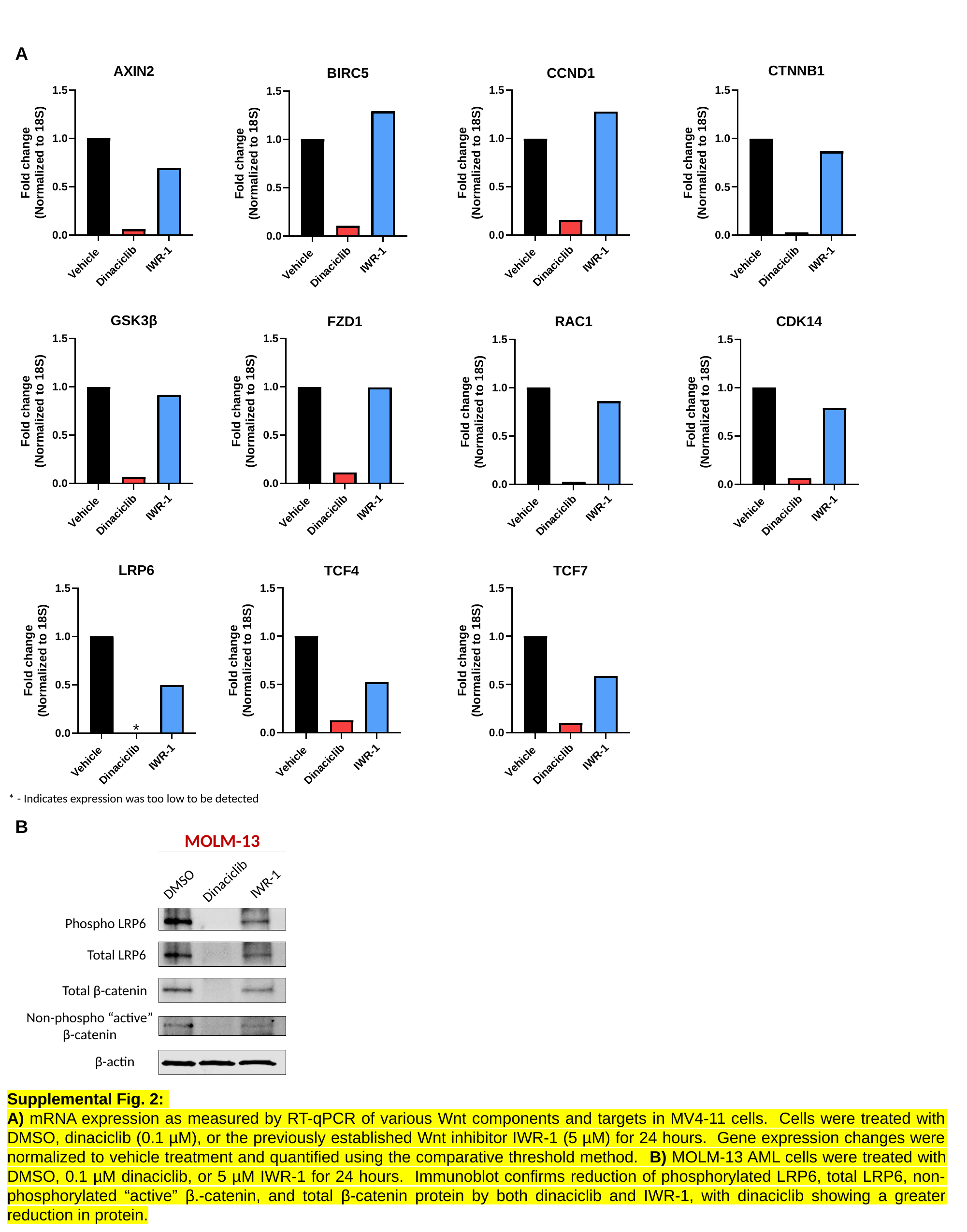

A
* - Indicates expression was too low to be detected
B
MOLM-13
IWR-1
Dinaciclib
DMSO
Phospho LRP6
Total LRP6
Total β-catenin
Non-phospho “active” β-catenin
β-actin
Supplemental Fig. 2:
A) mRNA expression as measured by RT-qPCR of various Wnt components and targets in MV4-11 cells. Cells were treated with DMSO, dinaciclib (0.1 µM), or the previously established Wnt inhibitor IWR-1 (5 µM) for 24 hours. Gene expression changes were normalized to vehicle treatment and quantified using the comparative threshold method. B) MOLM-13 AML cells were treated with DMSO, 0.1 µM dinaciclib, or 5 µM IWR-1 for 24 hours. Immunoblot confirms reduction of phosphorylated LRP6, total LRP6, non-phosphorylated “active” β.-catenin, and total β-catenin protein by both dinaciclib and IWR-1, with dinaciclib showing a greater reduction in protein.

## Slide 4
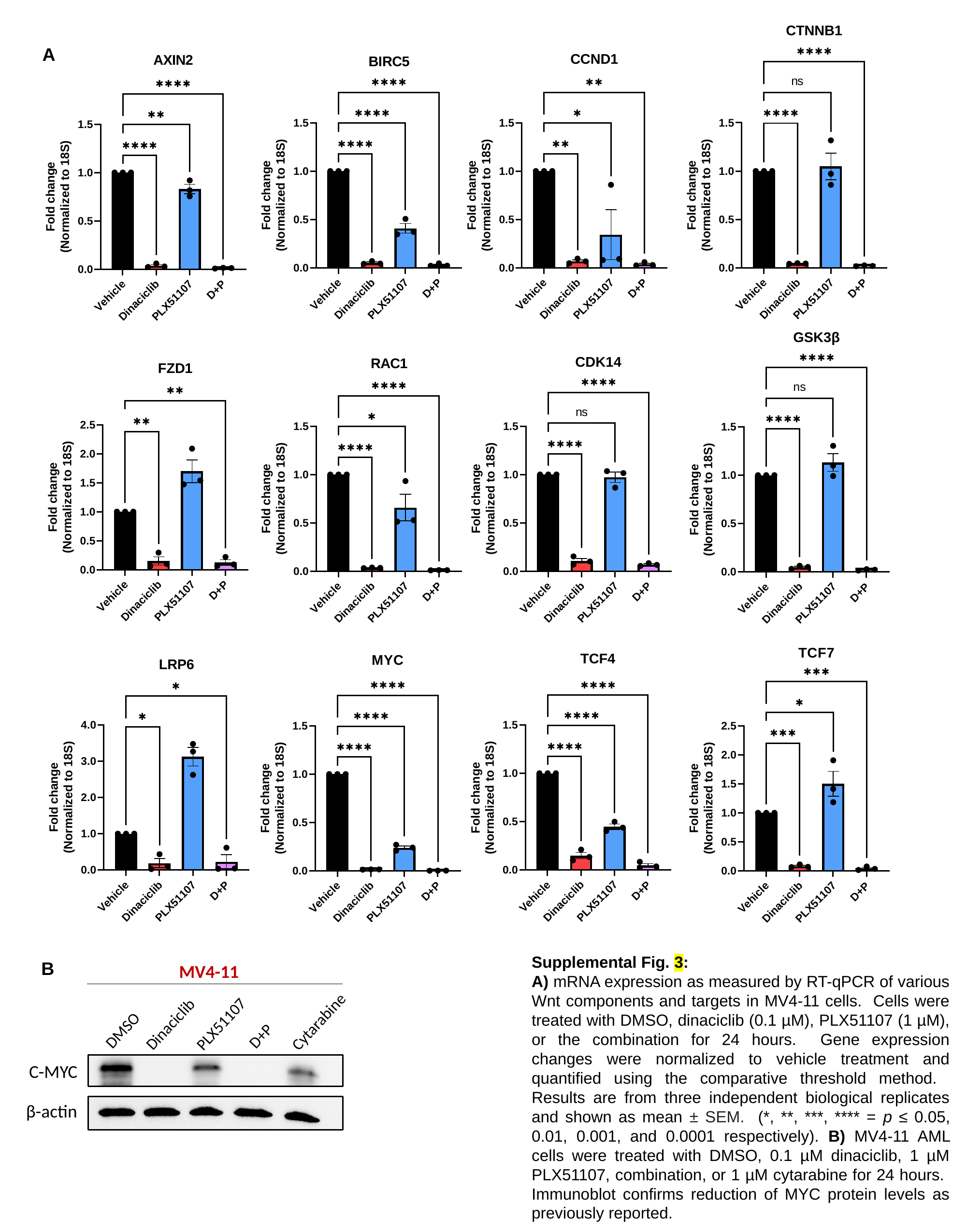

A
Supplemental Fig. 3:
A) mRNA expression as measured by RT-qPCR of various Wnt components and targets in MV4-11 cells. Cells were treated with DMSO, dinaciclib (0.1 µM), PLX51107 (1 µM), or the combination for 24 hours. Gene expression changes were normalized to vehicle treatment and quantified using the comparative threshold method. Results are from three independent biological replicates and shown as mean ± SEM. (*, **, ***, **** = p ≤ 0.05, 0.01, 0.001, and 0.0001 respectively). B) MV4-11 AML cells were treated with DMSO, 0.1 µM dinaciclib, 1 µM PLX51107, combination, or 1 µM cytarabine for 24 hours. Immunoblot confirms reduction of MYC protein levels as previously reported.
B
MV4-11
D+P
Dinaciclib
PLX51107
Cytarabine
DMSO
C-MYC
β-actin

## Slide 5
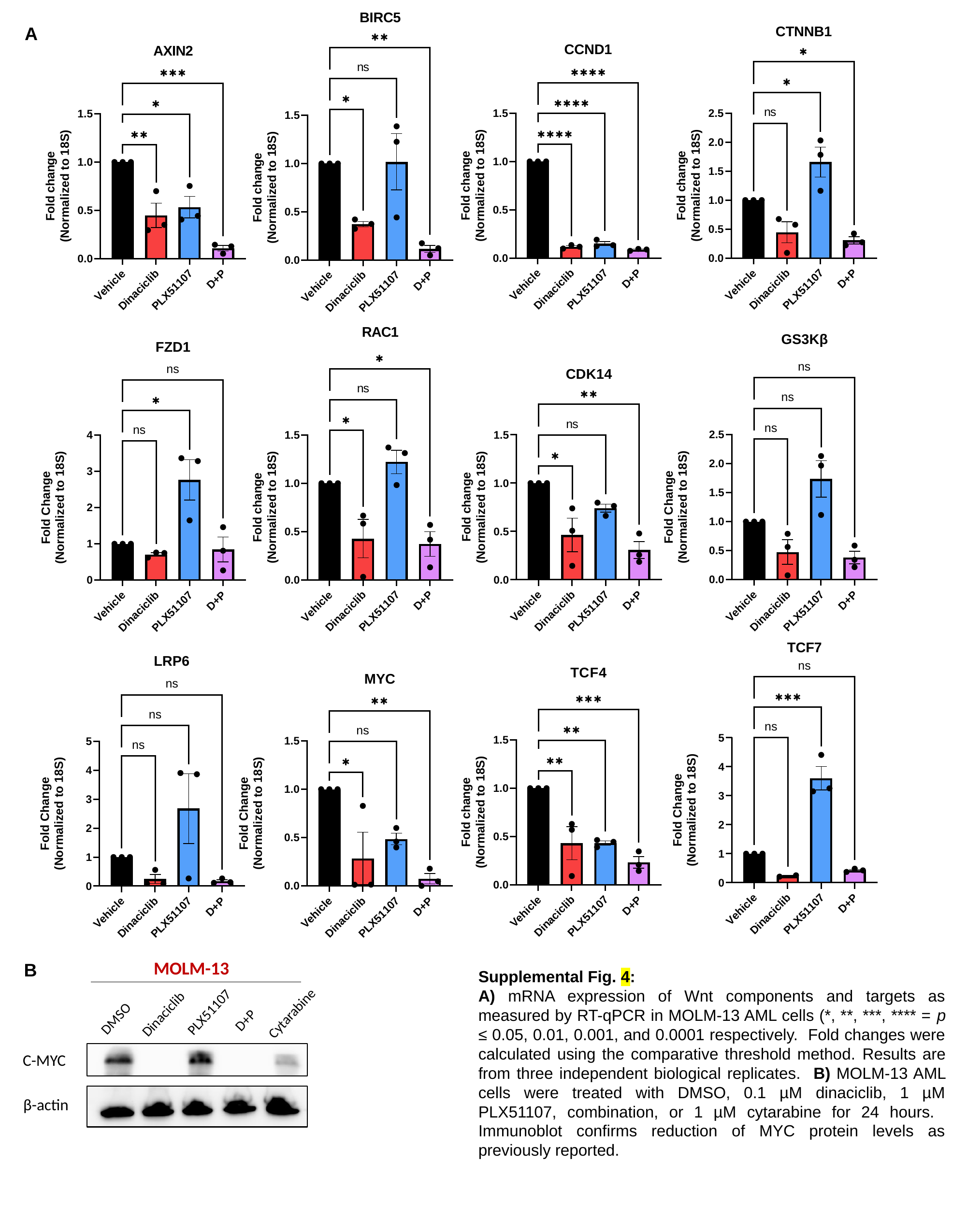

A
MOLM-13
D+P
PLX51107
Dinaciclib
Cytarabine
DMSO
C-MYC
β-actin
B
Supplemental Fig. 4:
A) mRNA expression of Wnt components and targets as measured by RT-qPCR in MOLM-13 AML cells (*, **, ***, **** = p ≤ 0.05, 0.01, 0.001, and 0.0001 respectively. Fold changes were calculated using the comparative threshold method. Results are from three independent biological replicates. B) MOLM-13 AML cells were treated with DMSO, 0.1 µM dinaciclib, 1 µM PLX51107, combination, or 1 µM cytarabine for 24 hours. Immunoblot confirms reduction of MYC protein levels as previously reported.

## Slide 6
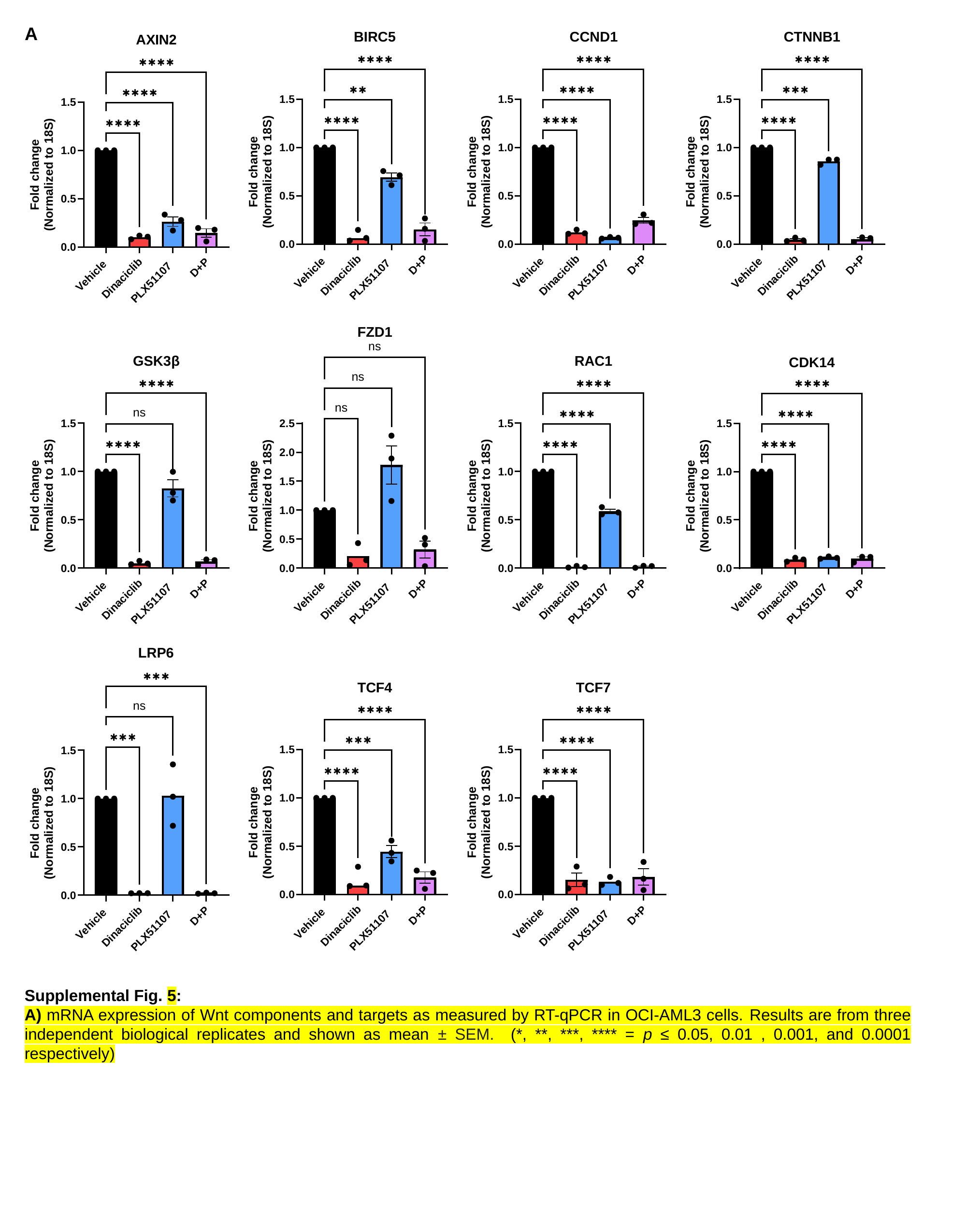

A
Supplemental Fig. 5:
A) mRNA expression of Wnt components and targets as measured by RT-qPCR in OCI-AML3 cells. Results are from three independent biological replicates and shown as mean ± SEM. (*, **, ***, **** = p ≤ 0.05, 0.01 , 0.001, and 0.0001 respectively)

## Slide 7
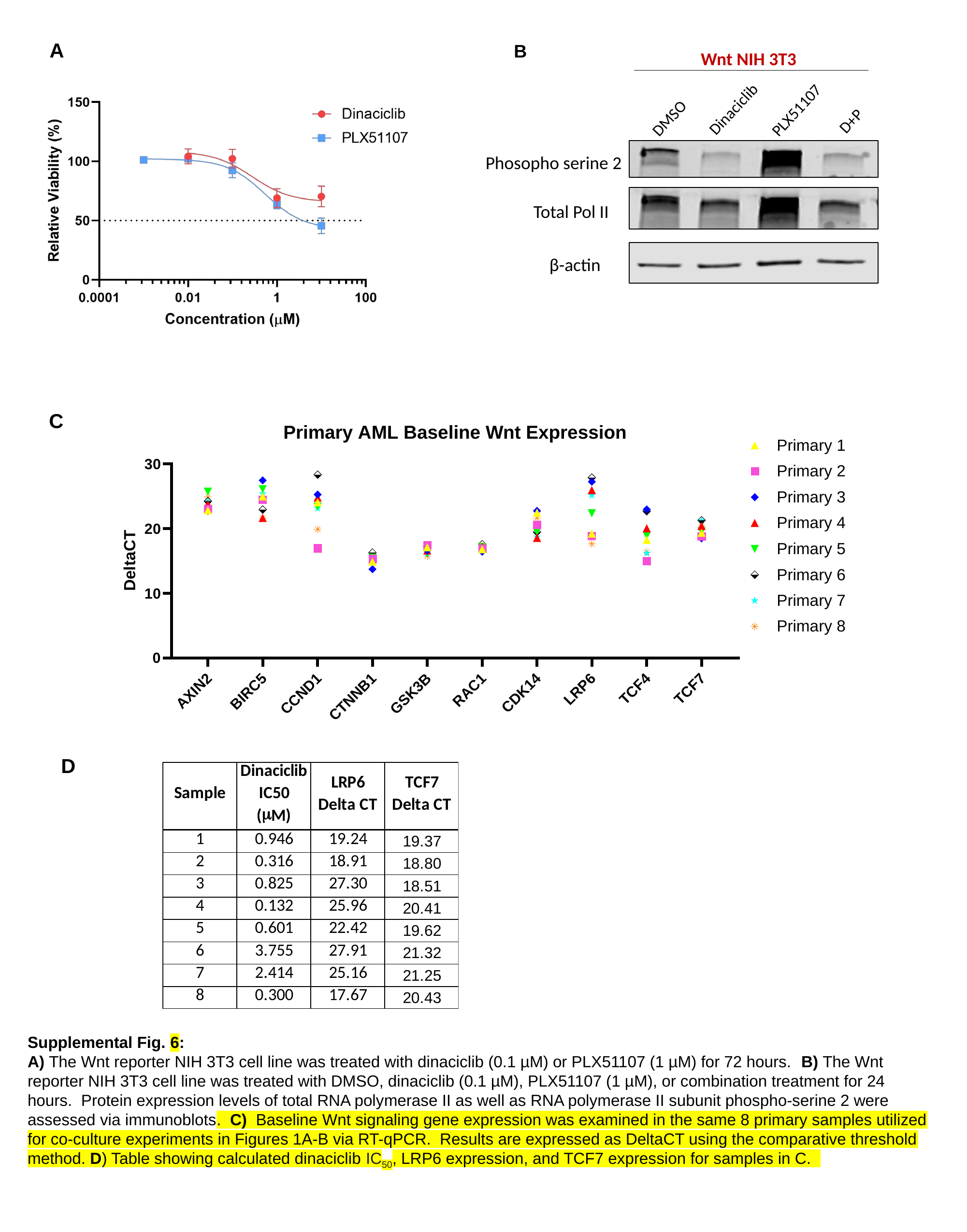

A
B
Wnt NIH 3T3
D+P
Dinaciclib
PLX51107
DMSO
Phosopho serine 2
Total Pol II
β-actin
C
D
Supplemental Fig. 6:
A) The Wnt reporter NIH 3T3 cell line was treated with dinaciclib (0.1 µM) or PLX51107 (1 µM) for 72 hours. B) The Wnt reporter NIH 3T3 cell line was treated with DMSO, dinaciclib (0.1 µM), PLX51107 (1 µM), or combination treatment for 24 hours. Protein expression levels of total RNA polymerase II as well as RNA polymerase II subunit phospho-serine 2 were assessed via immunoblots. C) Baseline Wnt signaling gene expression was examined in the same 8 primary samples utilized for co-culture experiments in Figures 1A-B via RT-qPCR. Results are expressed as DeltaCT using the comparative threshold method. D) Table showing calculated dinaciclib IC50, LRP6 expression, and TCF7 expression for samples in C.

## Slide 8
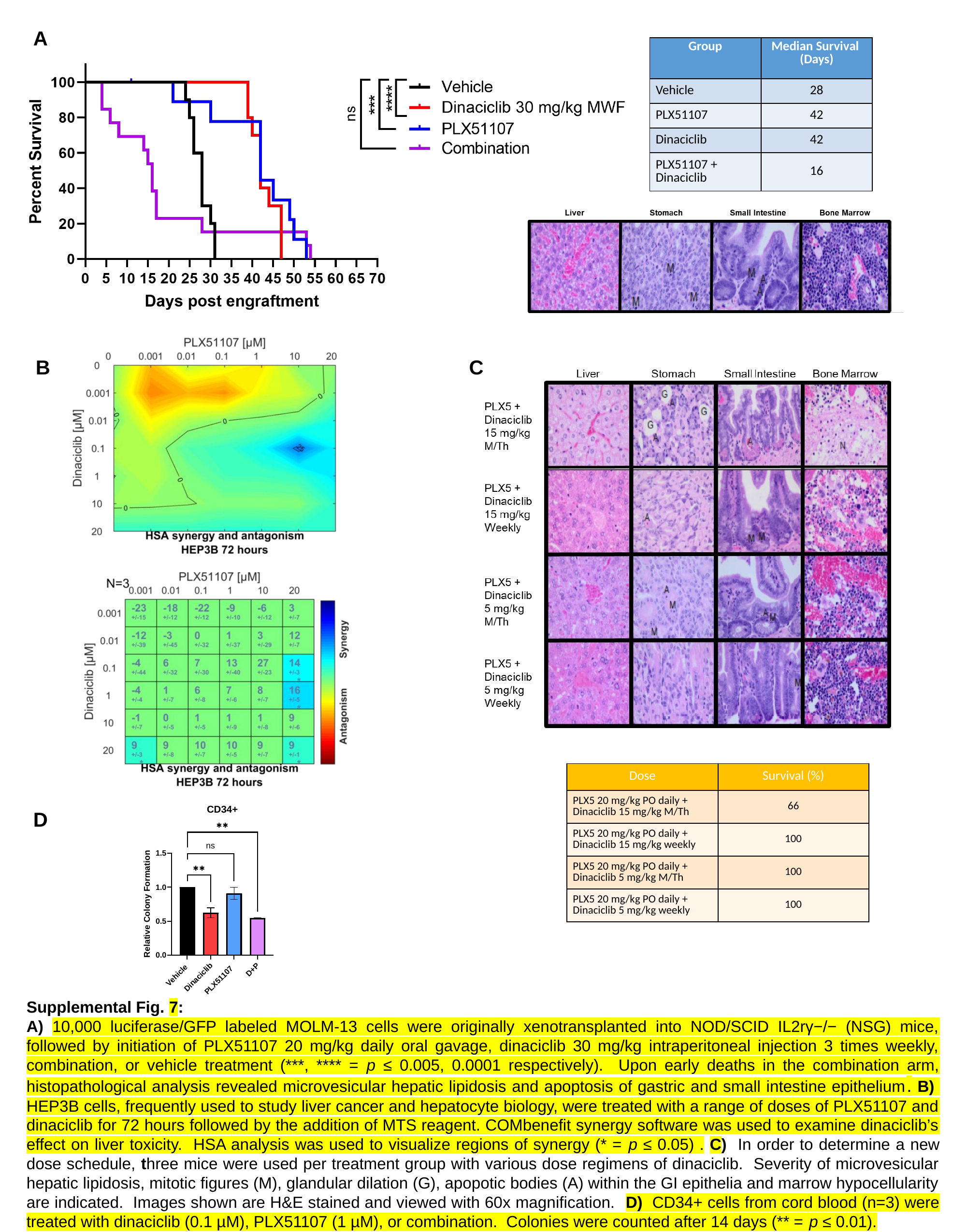

A
| Group | Median Survival (Days) |
| --- | --- |
| Vehicle | 28 |
| PLX51107 | 42 |
| Dinaciclib | 42 |
| PLX51107 + Dinaciclib | 16 |
B
C
| Dose | Survival (%) |
| --- | --- |
| PLX5 20 mg/kg PO daily + Dinaciclib 15 mg/kg M/Th | 66 |
| PLX5 20 mg/kg PO daily + Dinaciclib 15 mg/kg weekly | 100 |
| PLX5 20 mg/kg PO daily + Dinaciclib 5 mg/kg M/Th | 100 |
| PLX5 20 mg/kg PO daily + Dinaciclib 5 mg/kg weekly | 100 |
D
Supplemental Fig. 7:
A) 10,000 luciferase/GFP labeled MOLM-13 cells were originally xenotransplanted into NOD/SCID IL2rγ−/− (NSG) mice, followed by initiation of PLX51107 20 mg/kg daily oral gavage, dinaciclib 30 mg/kg intraperitoneal injection 3 times weekly, combination, or vehicle treatment (***, **** = p ≤ 0.005, 0.0001 respectively). Upon early deaths in the combination arm, histopathological analysis revealed microvesicular hepatic lipidosis and apoptosis of gastric and small intestine epithelium. B) HEP3B cells, frequently used to study liver cancer and hepatocyte biology, were treated with a range of doses of PLX51107 and dinaciclib for 72 hours followed by the addition of MTS reagent. COMbenefit synergy software was used to examine dinaciclib’s effect on liver toxicity. HSA analysis was used to visualize regions of synergy (* = p ≤ 0.05) . C) In order to determine a new dose schedule, three mice were used per treatment group with various dose regimens of dinaciclib. Severity of microvesicular hepatic lipidosis, mitotic figures (M), glandular dilation (G), apopotic bodies (A) within the GI epithelia and marrow hypocellularity are indicated. Images shown are H&E stained and viewed with 60x magnification. D) CD34+ cells from cord blood (n=3) were treated with dinaciclib (0.1 µM), PLX51107 (1 µM), or combination. Colonies were counted after 14 days (** = p ≤ 0.01).

## Slide 9
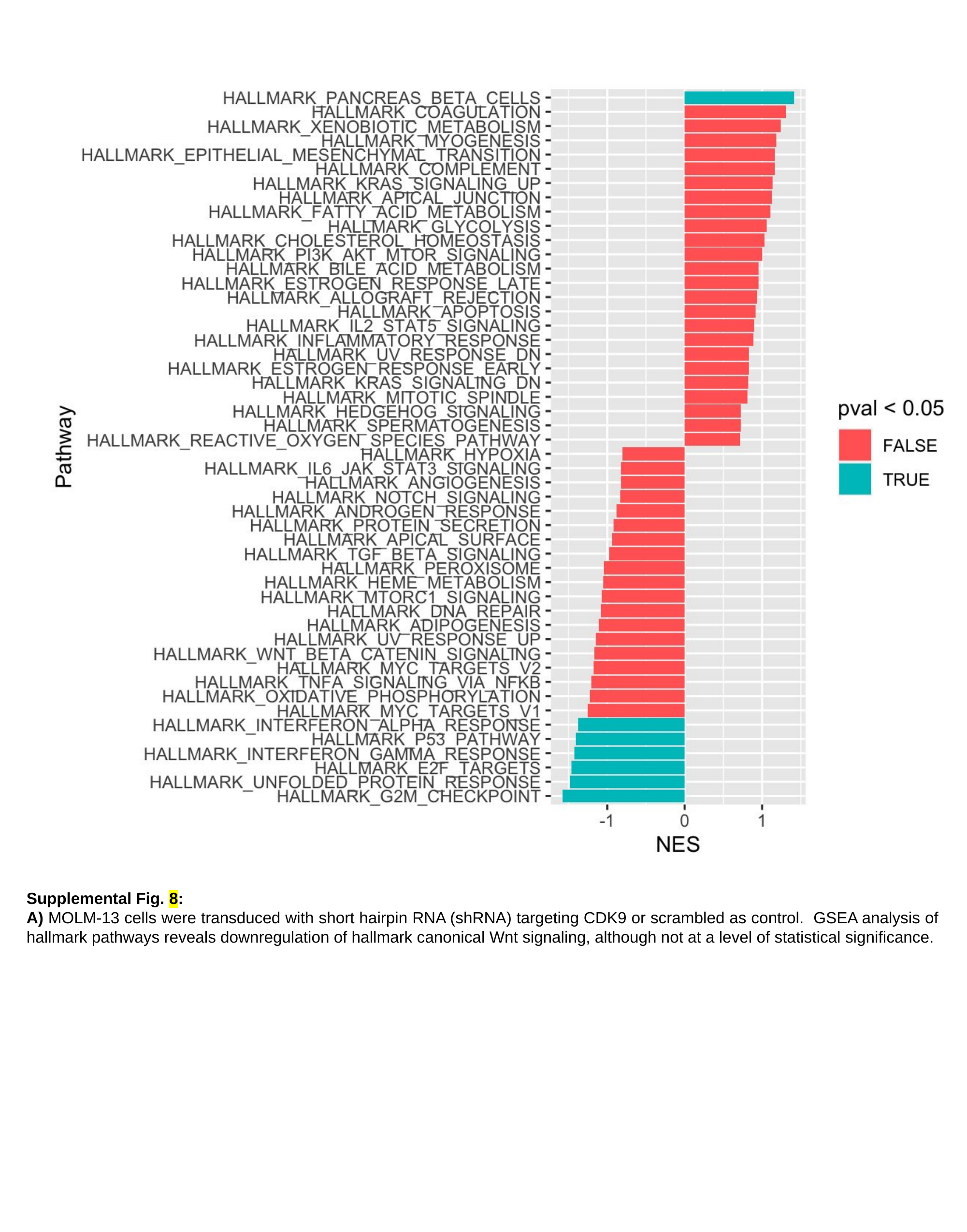

Supplemental Fig. 8:
A) MOLM-13 cells were transduced with short hairpin RNA (shRNA) targeting CDK9 or scrambled as control. GSEA analysis of hallmark pathways reveals downregulation of hallmark canonical Wnt signaling, although not at a level of statistical significance.
